# Exploring the Link Between Autophagy‐Lysosomal Dysfunction and Early Heterotopic Ossification in Tendons

**DOI:** 10.1002/advs.202400790

**Published:** 2024-05-13

**Authors:** Chang‐He Gao, Qian‐Qian Wan, Jan‐Fei Yan, Yi‐Na Zhu, Lei Tian, Jian‐Hua Wei, Bin Feng, Li‐Na Niu, Kai Jiao

**Affiliations:** ^1^ Department of Stomatology Tangdu Hospital State Key Laboratory of Oral & Maxillofacial Reconstruction and Regeneration School of Stomatology The Fourth Military Medical University Xi'an Shaanxi 710032 P. R. China; ^2^ State Key Laboratory of Oral & Maxillofacial Reconstruction and Regeneration & National Clinical Research Center for Oral Diseases & Shaanxi Key Laboratory of Stomatology School of Stomatology The Fourth Military Medical University Xi'an Shaanxi 710032 P. R. China; ^3^ Department of Stomatology The Third Affiliated Hospital of Xinxiang Medical University Xinxiang Henan 453000 P. R. China

**Keywords:** autophagy, heterotopic ossification, lysosome, pathological calcification, tendon injury

## Abstract

Heterotopic ossification (HO), the pathological formation of bone within soft tissues such as tendon and muscle, is a notable complication resulting from severe injury. While soft tissue injury is necessary for HO development, the specific molecular pathology responsible for trauma‐induced HO remains a mystery. The previous study detected abnormal autophagy function in the early stages of tendon HO. Nevertheless, it remains to be determined whether autophagy governs the process of HO generation. Here, trauma‐induced tendon HO model is used to investigate the relationship between autophagy and tendon calcification. In the early stages of tenotomy, it is observed that autophagic flux is significantly impaired and that blocking autophagic flux promoted the development of more rampant calcification. Moreover, Gt(ROSA)26sor transgenic mouse model experiments disclosed lysosomal acid dysfunction as chief reason behind impaired autophagic flux. Stimulating V‐ATPase activity reinstated both lysosomal acid functioning and autophagic flux, thereby reversing tendon HO. This present study demonstrates that autophagy‐lysosomal dysfunction triggers HO in the stages of tendon injury, with potential therapeutic targeting implications for HO.

## Graphical abstract

In the early stages of heterotopic ossification (HO) development, V‐ATPase dysfunction can lead to insufficient lysosomal acidification in damaged tendon tissue, which in turn hinders autophagy flux and accelerates tendon calcification. It is hypothesized that the accumulation of autophagosomes in damaged tendon tissue can facilitate the release of extracellular vesicles into the extracellular matrix, contributing to the formation of HO.

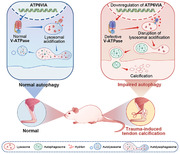

## 1 Introduction

Heterotopic ossification (HO) is defined as the formation of mature calcified bone in soft tissues such as skeletal muscle and tendons, which can also be referred to as ectopic calcification.^[^ ^1^ ^]^ In addition to causing pain due to chronic inflammation and tissue deformation, per‐articular HO limits joint mobility and limb function, thereby affecting patients’ quality of life. To date, clinical therapy for HO is limited to radiation, non‐steroidal anti‐inflammatory drugs (NSAIDs), and surgery with high recurrence rates.^[^ ^2^ ^]^ Tendinopathy is a prevalent condition in sports injuries, accounting for more than 50% of all such injuries that result in HO of the tendon.^[^ ^3^ ^]^ In general, 14%−62% of tendons develop ossification after percutaneous Achilles tendon repair, leading to tendinopathy progressing to tendon HO.^[^ ^4^ ^]^ Due to the lack of understanding of developing mechanism of HO, there are no effective methods to prevent or reverse the progression of tendon calcification and thereby protect the tendon from HO.

HO is well‐orchestrated process of ossification precisely mediated by multiple cells and molecules. Previous studies identified various molecules serving as causative agents of HO, including transforming growth factor‐β and bone morphogenetic protein type I receptor, the blocking of which can effectively mitigate HO progression.^[^ ^5^ ^]^ Usually, there are four stages involved during HO process: inflammation and primary calcification, chondrogenesis, osteogenesis of differentiated cells, and maturation of calcification into ossification.^[^ ^6^ ^]^ Primary calcification causes changes in the hardness of the extracellular matrix, further promoting inflammation and osteogenic differentiation of surrounding cells.^[^ ^7^, ^8^ ^]^ During the early stage of HO, formation of primary calcification has been reported to be related with release of extracellular vesicles.^[^ ^9^, ^10^ ^]^ It has been reported that cells within the pathological environment release vesicles containing precursor of calcification or proteins, which can deposit on the collagen matrix and promote pathologic calcification in cases of osteoarthritis and tendon injury.^[^ ^11^, ^12^ ^]^ However, the mechanisms underlying intracellular formation and release of these extracellular vesicles during HO are poorly understood.

During the process of ossification, autophagy plays a critical role in the differentiation, transformation and functional activity of key mineralizing cells such as osteoblasts and chondrocytes.^[^ ^13^ ^]^ Recent studies have shown that autophagy plays an important role in maintaining the homeostasis of intracellular mineral metabolism.^[^ ^14^ ^]^ Dysfunctional autophagy has also been reported to contribute to the development of a variety of pathological calcifications (diabetes mellitus, aortic valve calciphylaxis, chronic kidney disease, and chronic inflammatory diseases) through the release of matrix vesicles.^[^ ^15^, ^16^, ^17^, ^18^ ^]^ For example, our recent studies have shown that autophagic LC3 extracellular vesicles derived from chondrocytes mediate the development of pathological calcification in osteoarthritis and that restoration of autophagy is effective in halting the progression of calcification.^[^ ^11^ ^]^ Indeed, we have demonstrated the accumulation of autophagosomes early in tendon injury, but the relationship between autophagic dysfunction and the pathology of tendon calcification remains unclear.

The process of autophagy mainly involves the formation of autophagosomes and the fusion of autophagosomes with lysosomes to form autolysosomes.^[^ ^19^ ^]^ autophagosomes mature into autolysosomes after fusion with lysosomes or endolysosome, which introduces various cathepsin proteases, other acid hydrolases, and V‐ATPases, the proton pump that acidifies the autolysosome lumen and activates the hydrolases.^[^ ^20^ ^]^ Therefore, lysosomal dysfunction will disrupt the clearance of autophagy and may lead to the development of a variety of diseases. For example, structural abnormalities and impaired function of lysosome in skeletal muscle result in the accumulation of autophagosome in muscle fibers, leading to the progression of osteopetrosis and Danon disease.^[^ ^21^, ^22^, ^23^, ^24^ ^]^ However, the role of the autophagosome‐lysosome pathway in the regulation of tendon HO has not been elucidated.

In this study, a mouse model of trauma‐induced tendon HO was developed. In this model, it was observed that early tendon injury was accompanied by the accumulation of autophagosome. Based on this model and the above findings, the aim of this study was to investigate the causes of autophagy dysfunction and its contribution to tendon calcification. The results should provide physicochemical mechanistic insights into the role of autophagy in mediating tendon HO.

## 2 Results

### 2.1 Trauma ‐Induces Tendon Calcification with Progressive Aggravation in Mouse Model

**Figure 1:**
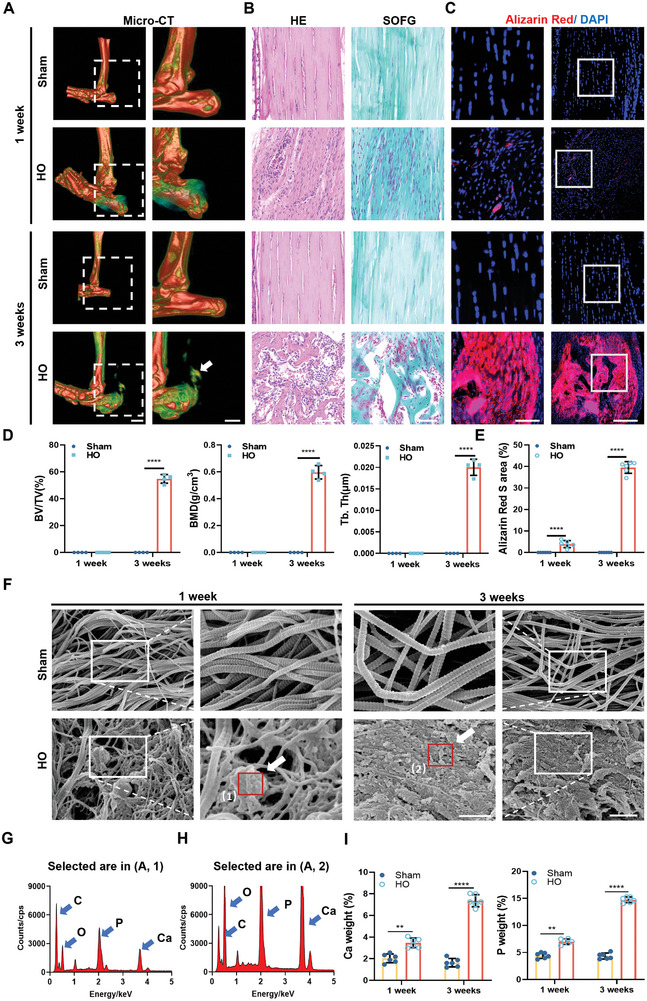
Trauma ‐induces tendon calcification with progressive aggravation in mouse model. A) Representative micro‐CT images of the Achilles tendon from sham groups of and HO groups mice at 1 and 3 weeks after surgery (Arrows denote tendon calcification) (*n* = 4). Scale bar: 2 mm. B) Representative H&E, SOFG images of the Achilles tendon from sham groups and HO groups mice at 1 and 3 weeks after surgery (*n* = 6). Scale bar: 50 µm. C) Representative confocal images of the Achilles tendon calcification sections stained with alizarin red S (red) and DAPI (blue) in sham groups of and HO groups mice at 1 and 3 weeks after surgery (*n* = 6). Scale bar, 100 µm. D) Quantitative analysis of bone volume (BV/TV), bone mineral density (BMD) and trabecular thickness (Tb.Th) (*n* = 4). E) Analysis of areas stained with Alizarin Red S in (C). Quantification of calcification was done with the entire percentage area of the entire tendon (*n* = 6). F) Representative SEM images of the Achilles tendon from sham and HO groups mice at 1 and 3 weeks after surgery (*n* = 6). Scale bar, 5 µm. Magnification of the area depicted by the white rectangle shows collagen fibers, calcified particles, and mineralized deposits (arrows). Scale bar, 200 nm. G,H) Elemental analysis of the calcified particles indicated by the arrows in (A). I) Quantitative analysis of element distribution in the the Achilles tendon (*n* = 6). Statistical analyses are performed by two‐way ANOVA with Holm‐Šidák multiple comparison tests. **P < 0.01, ****P < 0.0001.

To study the development of pathological calcification after tendon injury, a mouse model of trauma‐induced tendon HO was established according to the literature.^[^ ^1^ ^]^ At 1 and 3 weeks after surgery, HO was found locally in the Achilles tendon of 3‐week‐old HO mouse, examined by micro‐computed tomography (micro‐CT) (**Figure** **1**A,D). Histologically, H&E and SOFG staining showed the presence of bone trabeculae and bone marrow cavities at the Achilles tendon in 3‐week‐old HO mouse (Figure 1B). In addition, pathological calcifications stained red by alizarin red S were observed in the Achilles tendon of the mouse as early as 1 week after surgery. The condition worsened at 3 weeks after surgery (Figure 1C). The areas occupied by calcifications in the tendons of the HO groups were significantly increased compared with those of the sham groups (Figure 1E). To further investigate the process of pathological calcification of tendons in HO, ultrastructural and elemental analysis of tendons from the sham and HO groups were carefully examined. Enlarged SEM images of the Achilles tendon showed that the collagen fibers were disorganized and some of the lateral structure of collagen in the tendon was lost in the first week in the HO group compared with the sham group (Figure 1F). Elemental analysis showed that they contain both calcium and phosphate (Figure 1G). The condition worsened by the third week, with loss of collagen striations and extensive calcification on the surface of collagen fibers (Figure 1F,H). No calcification was found in any tendon sample from the sham group (Figure S1, Supporting Information). The relative amount of calcium and phosphorus in the tendons of both HO groups was much higher than in the sham groups (Figure 1I). In addition, we found a large amount of colocalization of Col‐I immunofluorescence staining and Alizarin Red staining (Figure S2, Supporting Information), which indicated that the formation of early calcification in tendon tissue mainly occurs in the collagen. In order to further study the microscopic morphology of tendon tissue, atomic force microscopy (AFM) was used to observe it. The sham group displayed regular and ordered collagen, whereas the HO group exhibited haphazardly arranged collagen, accompanied by deposits resembling calcified particles (Figure S3, Supporting Information). These results suggest that trauma induces calcification of the tendon, which increases as the condition worsens.

### 2.2 Impaired Autophagic Flux was Found in Injured Tendons

**Figure 2:**
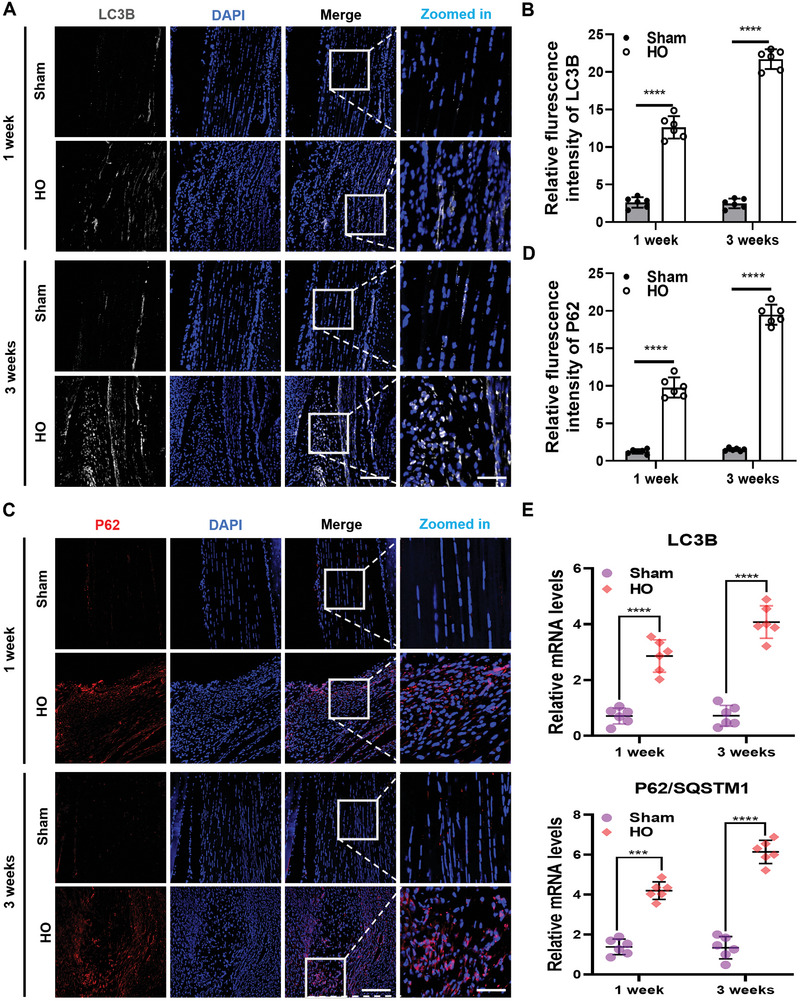
Impaired autophagic flux was found in injured tendons. A) Immunofluorescence staining of LC3B (gray) and DAPI (blue) in the Achilles tendon of mice in sham and HO groups at 1 and 3 weeks after surgery (*n* = 6). bar, 100 µm. B) Quantitative analysis of the fluorescence intensity of LC3B expression in different groups (*n* = 6). C) Representative confocal images of P62 (red) and DAPI (blue) immunostaining Achilles tendon in sham groups and HO groups mice at 1 and 3 weeks after surgery (*n* = 6). Scale bar, 100 µm. D) Quantitative analysis of the fluorescence intensity of P62 expression in different groups (*n* = 6). E) Quantitative real‐time polymerase chain reaction analysis of the gene expression of the autophagy‐related in mice Achilles tendon at different time points after surgery (*n* = 6). Statistical analyses are performed by two‐way ANOVA with Holm‐Šidák multiple comparison tests. ***P < 0.001, ****P < 0.0001.

Previous studies have shown that under conditions of tissue injury (brain injury, liver injury, pancreatic injury, kidney injury) or disease, autophagic flux may be impaired and unable to keep up with the production of autophagosome.^[^ ^25^, ^26^ ^]^ However, this has not been reported in tendon injury. To evaluate autophagic flux in injured tendons, we quantified markers of autophagy in the sham and HO groups. First, we detected high levels of LC3B (a classical marker of autophagy) in the tendons of the HO groups, suggesting that autophagy levels were increased in the injured tendons (**Figure** **2**A,B). Immunofluorescence staining showed that this phenomenon mainly appeared in the collagen region of the injured tendon tissue (Figure S4, Supporting Information). In addition, we used western blot to detect the LC3B‐II/LC3B‐I ratio (Figure S5, Supporting Information). The results showed that the content of LC3B‐II was significantly upregulated in the injured tendon tissue, indicating an increased accumulation of autophagosome in the tissue. To confirm the inhibition of autophagic flux, we tested the protein chelator 1 (SQSTM1/P62). SQSTM1/P62 is a protein that is chelated in the autophagosome and preferentially degraded by lysosome hydrolysis along with other substrates.^[^ ^27^ ^]^ Therefore, an increase in P62 protein abundance indicates that autophagic degradation is inhibited. At 1 and 3 weeks after surgery, we detected a significant increase in P62 protein expression in the tendons of the HO groups compared to those of the sham groups (Figure 2C,D). Furthermore, quantitative PCR showed that the expressions of autophagy markers, including SQSTM1/P62 and LC3B, were significantly higher in tendons from HO groups compared with those from sham groups (Figure 2E). Taken together, the experimental results indicate that autophagic flux is inhibited in a mouse model of trauma‐induced tendon early HO.

### 2.3 Impaired Autophagic Flux can Exacerbate the Progression of Tendon Calcification

**Figure 3:**
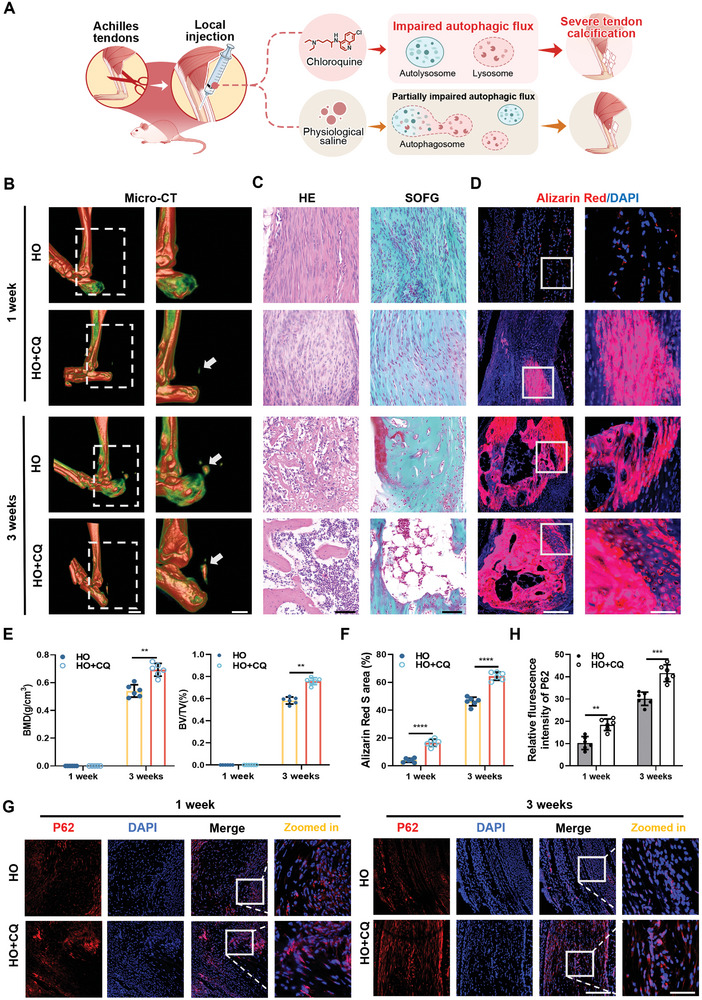
Impaired autophagic flux can exacerbate the progression of tendon calcification. A) Schematic diagram of chloroquine administration and tendon‐induced autophagosome accumulation. B) Representative micro‐CT images of the Achilles tendon from HO groups and HO+CQ groups mice at 1 and 3 weeks after surgery (Arrows denote tendon calcification) (*n* = 6). Scale bar: 2 mm. C) Representative H&E, SOFG images of Achilles tendon from HO groups and HO+CQ groups mice at 1 and 3 weeks after surgery (*n* = 6). Scale bar: 50 µm. D,F) Representative immunofluorescence staining and quantification of the Achilles tendon from HO groups and HO+CQ groups mice at different time points (*n* = 6). Scale bar, 100 µm. E) Quantitative analysis of bone mineral density (BMD) and bone volume (BV/TV) (*n* = 6). J,H) Representative immunofluorescence staining and quantitative analysis of local P62 protein in the Achilles tendon of different groups of mice (*n* = 6). Scale bar, 100 µm. Statistical analyses are performed by two‐way ANOVA with Holm‐Šidák multiple comparison tests. **P < 0.01, ***P < 0.001, ****P < 0.0001.

**Figure 4:**
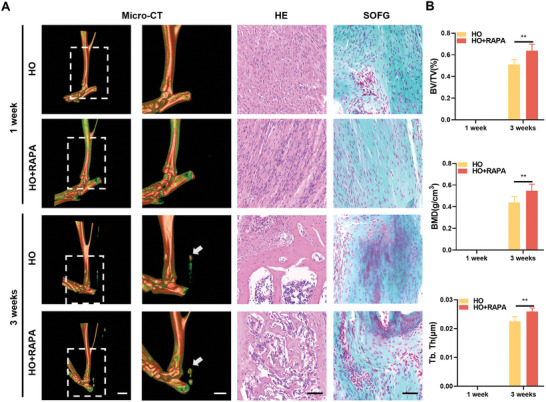
Increased autophagy flux does not alleviate the progression of tendon calcification. A) Representative micro‐CT images and H&E, SOFG images of the Achilles tendon at 1 week and 3 weeks after surgery from HO group and HO+RAPA groups and. Arrows denote heterotopic calcification. Scale bar: 2 mm, Scale bar: 50 µm. B) Quantitative analysis of bone volume (TV/BV), bone mineral density (BMD) and trabecular thickness (Tb.Th) (*n* = 3). Statistical analyses are performed by two‐way ANOVA with Holm‐Šidák multiple comparison tests. **P < 0.01.

To elucidate the relationship between autophagosome accumulation and tendon calcification, we blocked autophagic flux using chloroquine (CQ),^[^ ^28^ ^]^ which can inhibit the fusion of autophagosome and lysosome (**Figure** **3**A). CQ was injected subcutaneously into mouse immediately after tenotomy. HO was detected in mouse tendons in the HO+CQ groups as early as 1 week after surgery (Figure 3B). At 3 weeks after surgery, significantly increased HO formation was observed in HO+CQ groups compared to HO groups (Figure 3B,E) (Figure S6, Supporting Information). In addition, the same results were observed by H&E, Safranin O/fast green (SOFG), and alizarin red staining (Figure 3C,D,F). The results of the experiment showed that calcification occurred earlier in the tendons of the HO+CQ groups compared with the HO groups, and the degree of calcification was more severe. Consistent with the above results, the expression of LC3B was significantly increased in the tendons of the HO+CQ group compared with the HO group (Figure S6, Supporting Information). Immunostaining showed that the expression of P62 was also significantly increased in the HO+CQ group, indicating further accumulation of autophagosome in these tendons of the HO+CQ groups (Figure 3G,H). Additionally, we attempted to enhance autophagic flux by administering rapamycin (PAPA) to promote the fusion of autophagosomes and lysosomes, and we observed levels of tendon calcification.^[^ ^29^ ^]^ Analysis of micro‐CT results showed a significant increase in tendon tissue calcification in the HO+RAPA group compared to the control group at 1 week and 3 weeks after surgery (**Figure** **4**A,B). In addition, mCherry‐EGFP‐LC3 labeling showed that although RAPA promoted the fusion of autophagosomes and lysosomes, the use of RAPA did not mitigate the obstruction of autophagy flux in the HO+RAPA group (Figure S7, Supporting Information). Collectively, these results suggest that the obstruction of autophagy flux is an important factor in accelerating tendon calcification.

### 2.4 Tendon Damage Leads to Impaired Autophagic Flux but does not Affect the Fusion of Autophagosome with Lysosome

**Figure 5:**
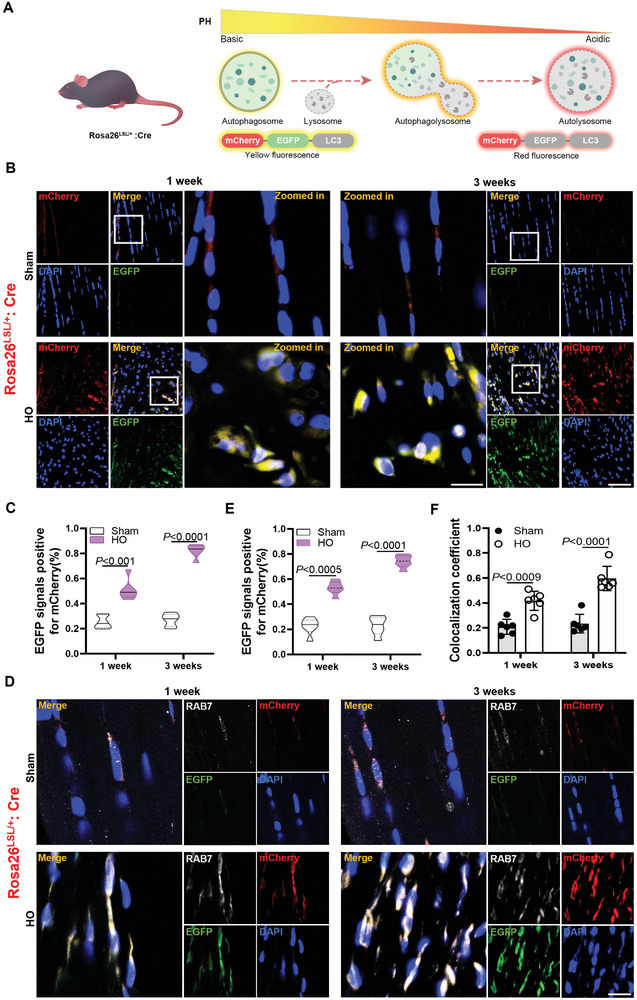
Tendon damage leads to impaired autophagic flux but does not affect the fusion of autophagosome with lysosome. A) Schematic representation of the color change of autophagosomes in genetic mouse cells. The autophagosome comprises of pH‐tolerant EGFP and mCherry. The acidic environment triggers quenching of the EGFP signal to switch from a yellow signal to a red‐only signal. B) Representative images of local fluorescence changes in the Achilles tendon from sham groups and HO groups mice at 1 and 3 weeks after surgery (*n* = 6). Scale bar, 50 µm. C) Quantification of fluorescence intensity on mCherry relative to EGFP in the bar graph (*n* = 6). D–F) Representative local fluorescence images of the Achilles tendon from sham groups and HO groups mice at 1 and 3 weeks after surgery, co‐labeled and quantified with the autophagosome and lysosomal anchorage‐associated protein Rab7 (*n* = 6). Scale bar, 25 µm. Statistical analyses are performed by two‐way ANOVA with Holm‐Šidák multiple comparison tests.

Next, we investigated the causes of autophagic dysfunction. A tandem mCherry‐EGFP‐LC3 transgene (Gt(ROSA)26sor) driven by the CAG promoter is expressed in postnatal tendon tissue (Figure S8, Supporting Information). The fluorescence signal of both co‐expressions varies according to the acidic environment of the autophagosome within the cell. mCherry is stable in an acidic environment, whereas EGFP undergoes quenching in an acidic environment within the lysosome. This property allows cellular autophagy to be distinguished depending on whether the cell's own lysosome is functioning or not. In autophagosome at higher pH, EGFP fluoresces yellow in superposition with mCherry, whereas in lysosome at lower pH, EGFP is quenched and only a red fluorescence signal can be detected (**Figure** **5**A).

Trauma‐induced tendon HO could be equally developed in both Gt (ROSA)26sor transgenic mice and wild‐type mice. The aim to establish HO in Gt (ROSA)26sor transgenic mice is to dynamically evaluate the autophagic flux and lysosome function in vivo. After modeling with the Gt (ROSA)26sor transgenic mouse, we found a high yellow fluorescence signal in the HO group by immunostaining, indicating the accumulation of autophagosome and autolysosome in the injured tendons (Figure 5B,C). The results further confirm that tendon injury leads to blocked autophagic flux. This could be caused by two reasons: one is that autophagosome and lysosome fusion is blocked, and the other is that lysosome function is impaired.

To verify the formation of autolysosome, co‐localization of autophagosome with lysosome ‐anchored Ras‐related protein 7 (Rab7), a GTPase, was examined to suggest fusion of autophagosome with lysosome. The yellow fluorescent signal and Rab7 showed strong co‐localization in the HO group, indicating the formation of autolysosome (Figure 5D–F). These results demonstrate that dysfunctional autophagy in injured tendons does not affect autophagosome formation. To further verify the formation of autolysosome, we examined the gene expression of autophagosome‐ lysosome fusion‐related factors by quantitative PCR. Gene expression of autophagosome‐lysosome fusion‐related factors [SNAP29 (synaptosome associated protein 29), STX17 (syntaxin protein 17) and VAMP8 (vesicle associated membrane protein 8)] was significantly upregulated in the HO group compared with the sham group (Figure S9, Supporting Information). Furthermore, co‐localization of the yellow fluorescent signal with lysosomal‐associated membrane protein 1 (LAMP1) further confirmed these results. (Figure S10, Supporting Information). Taken together, these results indicate that tendon damage leads to impaired autophagic flux but does not affect the fusion of autophagosome with lysosome.

### 2.5 Impaired Autophagic Flux is Caused by the Disruption of the Acidic Environment of the Lysosome

**Figure 6:**
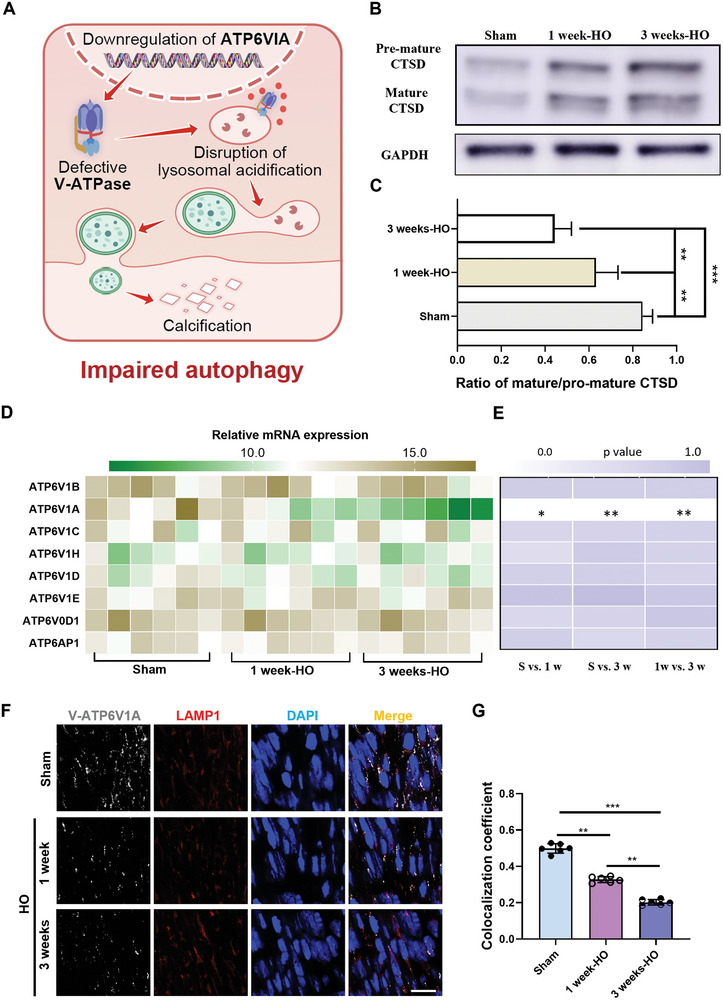
Impaired autophagic flux is caused by the disruption of the acidic environment of the lysosome. A) Diagram of the mechanism of lysosomal damage. B) Western blot analysis of CTSD protein in the Achilles tendon from sham groups and HO groups wild‐type mice at 1 and 3 weeks after surgery. C) The ratio of mature‐CTSD to premature‐CTSD was quantified in the bar graph (*n* = 3). D) Quantitative real‐time polymerase chain reaction analysis of the gene expression of V‐ATPase enzyme‐related factors in the Achilles tendon from sham groups and HO groups wild‐type mice at 1 and 3 weeks after surgery (*n* = 6). E) Quantitative analysis of differentially expressed genes in (B). F) Representative double‐labeled immunofluorescence microscopy of Achilles tendon from sham groups and HO groups wild‐type mice showing co‐localization of V‐ATP6V1A (grey), LAMP1 (red), and DAPI (blue) at 1 and 3 weeks after surgery (*n* = 6). Scale bar, 60 µm. (D) Quantitative analysis of the percentage of V‐ATP6V1A/LAMP1 co‐localization in the different groups (*n* = 6). Statistical analyses were performed using one‐way ANOVA with Holm‐Šidák multiple comparison tests. *P < 0.05, **P < 0.01, ***P < 0.001.

As described previously, in mCherry‐EGFP‐LC3 transgenic mice, the fluorescence signal can be affected by the acidity of the environment of autophagosomes within the cell. In acidic environment, mCherry remains stable, while EGFP is degraded. Therefore, only the red fluorescence signal is detected in autophagosomes‐bound lysosomes with low pH. When lysosomes are in high pH, the EGFP and mCherry signals together to produce yellow fluorescence. This feature allows us to evaluate lysosome function in the cell. Using these transgenic mice, we found a significant number of yellow‐fluorescent signals, indicating the abnormal acidic function of lysosomes in local tissues after Achilles tendon injury, which is the main cause of autophagosome accumulation.^[^ ^30^, ^31^ ^]^ Next, we further investigated lysosome's acidification capability, which is crucial for lysosomal enzymes maturation and activation. The HO group exhibited higher levels of early maturing histone proteases, as well as lower levels of mature CTSD, compared to the control group, suggesting an inhibition of the transition from the early to the mature form (**Figure** **6**B,C). The results further suggest that lysosome's capacity for acidification is reduced.

The acidic pH of the lysosome is primarily produced by the multimeric protein complex V‐ATPase.^[^ ^32^ ^]^ This complex, which serves as an ATP‐dependent proton pump, is present and functional in nearly all eukaryotic cells (Figure 6A). V‐ATPase utilizes ATP hydrolysis to actively transport protons to the lumen, creating and sustaining the acidic environment of the lysosome. Furthermore, V‐ATPase includes two subcomplexes: V0 (membrane‐bound) and V1 (cytoplasmic). Therefore, the activity of V‐ATPase was investigated in various tendon groups. Polymerase chain reaction analysis was conducted for detecting molecular changes in V‐ATPase in tendons, The two subcomplexes of V‐ATPase's major subunits were analyzed. The results showed that the expression of a related factor, V‐ATP6V1A, was significantly downregulated in the HO group as compared to the sham group (Figure 6D,E). To confirm this finding, we conducted immunofluorescence staining and observed that the V1A subunit of V‐ATPase exhibited a strong co‐localization with the lysosomal membrane protein LAMP1 in the sham group. However, in the HO group, this co‐localization was weak (Figure 6F,G). These results suggest that there is a disruption in the acidification function of lysosomes, which could be the primary cause of the blocked autophagic fluxes.

### 2.6 Damage of Autophagy‐Lysosome System in the Fibroblasts Calcification Model In Vitro

**Figure 7:**
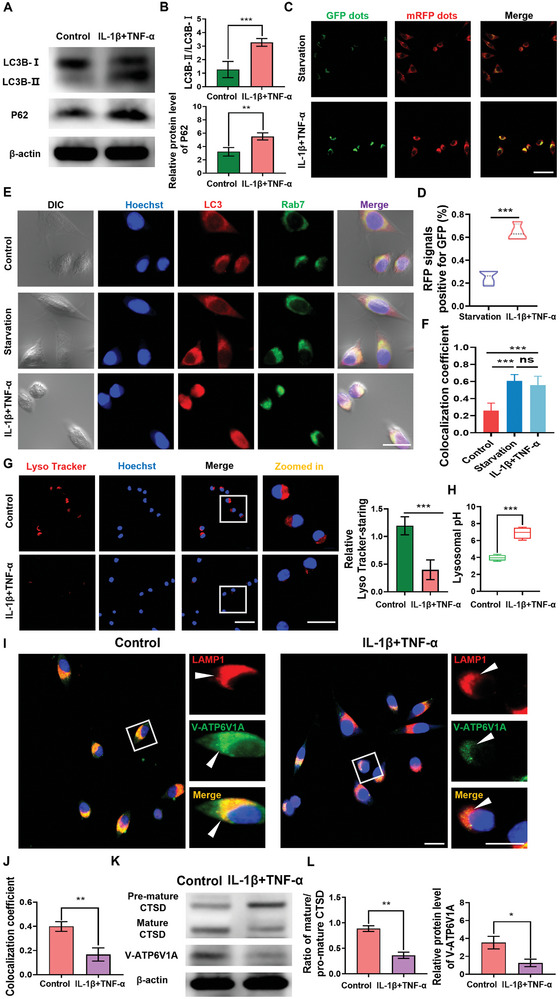
Damage of autophagy‐lysosome system in the fibroblasts calcification model in vitro. A) Western blot analysis of autophagy associated proteins (SQSTM1/P62, LC3B‐II/LC3B‐I) in control group and IL‐1β+TNF‐α group. B) The ratio of LC3B‐II to LC3B‐I and p62 protein level were quantified in the bar graph (*n* = 3). C) Representative immunofluorescence images of tendon fibroblasts that were transduced with mRFP‐GFP‐LC3 adenoviral vector and cultured under starvation condition or IL‐1β+TNF‐α group treatment (*n* = 3). D) Relative GFP fluorescence intensity over RFP was quantified in the bar graph (n = 3) Scale bars: 50 µm. E) Immunostaining with antibodies against Rab7 and LC3. Cells were starved, or treated with or without IL‐1β+TNF‐α. Scale bar: 25 µm. F) The colocalization coefficient of RAB7 and LC3 was represented as percentage of punctate signals of LC3 that were positive for RAB7 (*n* = 3). G) Control and IL‐1β+TNF‐α cells were stained with Lysotracker Red and Lysotracker dots were quantified (*n* = 3). Scale bar, 20 µm. H) Lysosomal pH was determined in Control and IL‐1β+TNF‐α cells. I) Representative immunofluorescence images show V‐ATPase V1A and LAMP1 in Cell which were treated with or without IL‐1β+TNF‐α, and then stained with antibodies against V‐ATPase V1A and LAMP1. Scale bars: 10 µm. J) The colocalization coefficients of V‐ATPase V1A and LAMP1 were quantified as percentage of punctate signals of V‐ATPase V1A that were positive for LAMP1 (*n* = 3). K) Western blot analysis of CTSD protein and V‐ATPase V1A which had been treated with IL‐1β+TNF‐α. L) The ratio of mature CTSD to immature CTSD and the expression of V‐ATPase V1A were quantified in the histogram (*n* = 3). Statistical analyses were performed using Student's *t*‐test in (B, D, H, J, L), and one‐way ANOVA with Holm‐Šidák multiple comparison tests in (F). NS, no significance; *P < 0.05, **P < 0.01, ***P < 0.001.

HO is a sophisticatedly orchestrated ossification process mediated by multiple cells and molecules, involving four stages: inflammation and primary calcification, chondrogenesis, osteogenesis, and bone maturation. Chondrocytes and osteoblasts play a crucial role in the process of ossification, but they mainly participate in the advanced stage of HO.^[^ ^9^ ^]^ However, we are still unclear about the initiating factors of early‐stage primary calcification. In the present study, we found that autophagy flux was blocked in injured tendon tissue and involved in the occurrence of local primary calcification. Previous studies demonstrated that fibroblasts play an important role in the early‐stage pathological calcification of tendon.^[^ ^33^, ^34^ ^]^ In addition, in the early stages of tendon injury, fibroblasts are activated and proliferate to promote tissue repair.^[^ ^35^ ^]^ Labeling of α‐SMA in mCherry‐EGFP‐LC3 mice (Figure S11, Supporting Information) revealed a very high amount of co‐labeling. This result indicated that in the early stage of tendon injury, fibroblasts were mainly involved in the event of autophagy flux blocking. To further explore the reasons for the obstruction of autophagy flux, we established an in vitro model of fibroblast calcification under the stimulation of IL‐1β and TNF‐α (Figures S12–S13, Supporting Information). We detected the expression levels of LC3B‐I/LC3B‐II and P62 using Western blot (**Figure** **7**A). The experimental results showed that the expression of LC3II and p62 was increased in the IL‐1β and TNF‐α group compared to the control group, indicating that autophagy flux was blocked (Figure 7B). In addition, we further examined autophagy flux by transferring the mRFP‐GFP‐LC3 fusion gene via an adenoviral vector. Under starvation conditions, autophagy is enhanced, and autophagosomes fuse with lysosomes to form a red fluorescence signal, mainly because GFP signals are easily quenched in acidic environments. However, in the IL‐1β and TNF‐α group, autophagy was enhanced and accompanied by a strong yellow fluorescence signal, indicating a significant decrease in the quenching of GFP fluorescence (Figure 7C,D). The results indicate that the degradation of autophagosomes is inhibited in the fibroblasts calcification model in vitro, and the autophagic flux is hindered. In order to further explore the reasons for the obstruction of autophagy flux, we found through immunofluorescence staining that a stronger co localization of LC3 and Rab7 in the IL‐1β and TNF‐α group compared to the control group, indicating that autophagosomes and lysosomes can form autophagosomes in the IL‐1β and TNF‐α group, and the degradation dysfunction of lysosomes may be the main reason for the obstruction of autophagy flux (Figure 7E,F). Therefore, we investigated the acidification function of lysosomes. We found through Lyso Tracker staining that the acidification ability of the IL‐1β and TNF‐α group lysosomes was decreased (Figure 7G,H). In addition, we found that the expression of V‐ATPeas enzyme subunit was downregulated in the IL‐1β and TNF‐α group compared to the control group (Figure 7I–L). In summary, the decrease in lysosomal acidity in the IL‐1β and TNF‐α group may be the main reason for the inhibition of autophagy flux.

### 2.7 Blocked Autophagy Flux Facilitates Release of Extracellular Vesicles to Promote Extracellular Matrix Calcification

**Figure 8:**
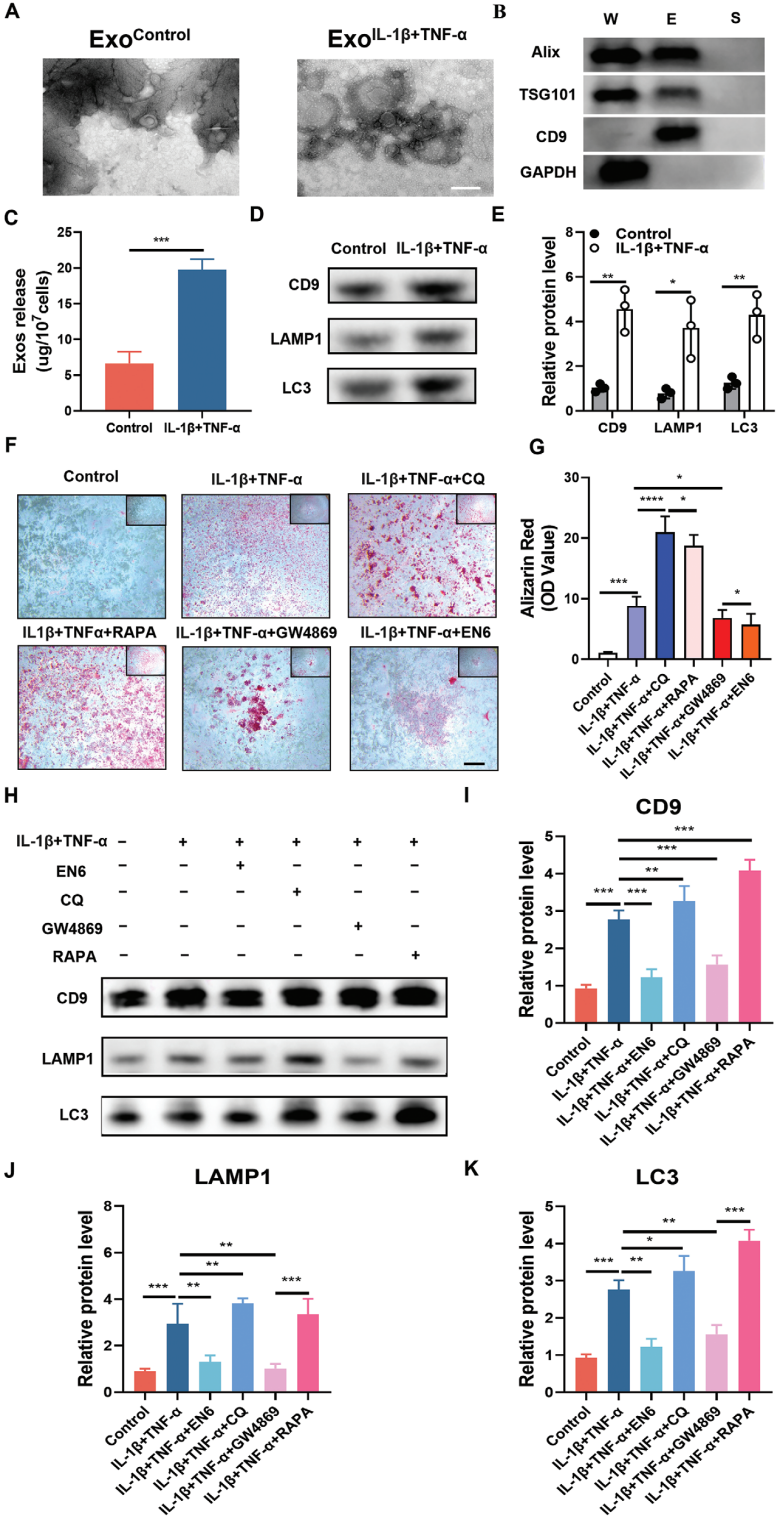
Blocked autophagy flux facilitates release of extracellular vesicles to promote extracellular matrix calcification. A) Representative TEM images of extracellular vesicles from tendon fibroblasts. Scale, 200 nm. B) Western blots analysis of Alix, TSG101, CD9, and GAPDH in the whole cell lysate (W), purified extracellular vesicles (E) from the conditioned medium of tendon fibroblasts, and supernatants after ultracentrifugation (S). C) Total protein was assessed in the extracellular vesicles that were purified from the conditioned medium of tendon fibroblasts treated with or without IL‐1β+TNF‐α. D) Western Blot analysis for detection of CD9, LC3, and LAMP1 expression in the purified extracellular vesicles. E) Relative protein levels were quantified in comparison with the control (*n* = 3). F) Alizarin red staining for calcification detection in tendon fibroblasts that were with IL‐1β+TNF‐α or/and EN6 or/and CQ or/and RAPA or/ and GW4869 for 14 days. G) The bar graphs show the quantification of Alizarin red in the cells (n = 3). H) Western blot analysis of CD9, LC3B and LAMP1 (*n* = 3). (I, J, and K) Relative protein levels were quantified in comparison with the control (*n* = 3). Statistical analyses were performed using Student's *t*‐test in (C), two‐way ANOVA with Holm‐Šidák multiple comparison tests in (E), and one‐way ANOVA with Holm‐Šidák multiple comparison tests in (G, I, J, K). *P < 0.05, **P < 0.01, ***P < 0.001, ****P < 0.0001.

Our previous work has found that LC3‐positive extracellular vesicles can promote pathological calcification owning to failure of binding between autophagosomes and lysosomes.^[^ ^11^ ^]^ These extracellular vesicles contain some pro‐calcification components, which are degraded by lysosomes in normal conditions. Therefore, we assume that the accumulated autophagosomes can be expelled by cells in the form of extracellular vesicles, which may also contain pro‐calcification components and mediate calcification in the extracellular matrix. To confirm this, extracellular vesicles were harvested and characterized (**Figure** **8**A,B) (Figure S14, Supporting Information).

More extracellular vesicles were harvested from the IL‐1β and TNF‐α group compared to the control group (Figure 8C). Next, to investigate the contribution of autophagy flux obstruction to calcification and its impact on secretion of extracellular vesicles, we regulated autophagic flux and observed the level of calcification as well as secretion of extracellular vesicles (Figure 8D–F). Through using autophagy inhibitor CQ and autophagy promoter RAPA, we found that the CQ/RAPA group had significantly increased levels of calcification compared to the control group, and the release of extracellular vesicles were significantly increased (Figure 8F,H). The extracellular vesicles specific protein CD9 and autolysosome associated LC3 and LAMP1 were relatively increased in the IL‐1β and TNF‐α group (Figure 8I–K). On the contrary, when we added the neutral sheath phospholipase (nSase) inhibitor GW4869 to block the release of extracellular vesicles, the extracellular vesicles harvested from the experimental group were significantly reduced. The extracellular vesicles specific protein CD9 and AL associated LC3 and LAMP1 were reduced in the IL‐1β and TNF‐α group (Figure 8I–K). In addition, we attempted to restore the acidification ability of lysosomes to observe their contribution to release of extracellular vesicles and calcification. When EN6 was added, we found that the extracellular vesicles specific protein CD9 and autolysosomes associated LC3 and LAMP1 were reduced in the IL‐1β and TNF‐α group, and the level of extracellular matrix calcification was significantly impaired (Figure 8F,I–K). In summary, these data suggest that inhibition of autophagy flux may promote calcification through enhancing release of extracellular vesicles.

### 2.8 Topical Treatment to Restore Lysosomal Acidification Capacity can Attenuate HO

**Figure 9:**
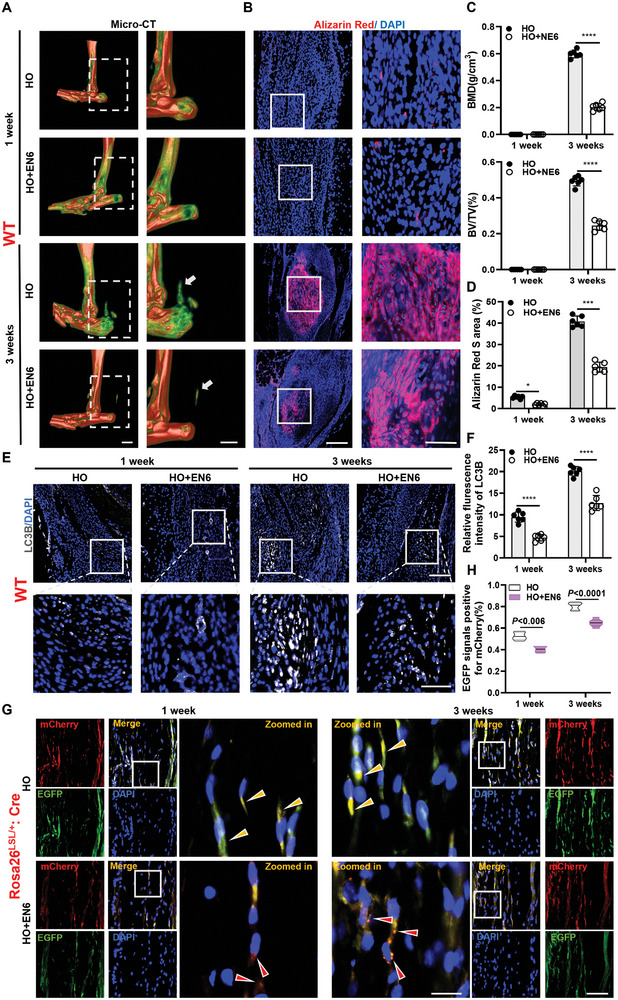
Topical treatment to restore lysosomal acidification capacity can attenuate HO. A,C) Representative micro‐CT images of calcification of the Achilles tendon from the wild‐type mice in HO groups and HO+NE6 groups. tendon calcification (arrows) at 1 and 3 weeks after surgery and quantitative analysis (*n* = 6). Scale bar: 2 mm. B,D) Representative immunofluorescence staining and quantification of the Achilles tendon from HO groups and HO+NE6 groups wild‐type mice at different time points (*n* = 6). Scale bar: 50 µm. E,F) Representative immunofluorescence staining of local LC3 protein and quantification of the Achilles tendon from HO groups and HO+NE6 groups wild‐type mice at different time points. LC3, grey; DAPI, blue (*n* = 6). Scale bar, 30 µm. G) Representative images of local fluorescence changes in Achilles tendon from HO groups and EN6 groups of transgenic mice (*n* = 6). Scale bar, 50 µm. impaired autophagic flux (yellow arrows) and normal autophagic flux (red arrows). H) Quantification of relative to EGFP fluorescence intensity on mCherry in a bar graph (*n* = 6). Statistical analyses are performed by two‐way ANOVA with Holm‐Šidák multiple comparison tests. *P < 0.05, ***P < 0.001, ****P < 0.0001.

Next, we investigated if restoring the acidic function of lysosome in injured tendons can reduce the development of HO. Previous research has suggested that NE6 boosts autophagosome‐lysosome fusion (autophagic flux) by targeting the V1A subunit activity of V‐ATPase, which then restores lysosomal re‐acidification.^[^ ^36^ ^]^ To evaluate the impact of EN6 on local autophagic function recovery and HO development in mouse tendons, we administered NE6 injections into injured tendons of wild‐type (WT) mouse immediately after tenotomy. At 1 and 3 weeks after surgery, HO was reduced significantly in mouse tendons in the HO+EN6 group compared to the HO group (**Figure** **9**A,C). Additionally, the area of calcification was significantly decreased in the HO+EN6 group compared to the HO group (Figure 9B,D). In addition, the expression level of LC3B and P62 was significantly lower in the Achilles tendon of WT mouse in the HO+EN6 group compared to the sham group, suggesting restoration of autophagic flux (Figure 9E,F) (Figure S15, Supporting Information). The results observed in the Achilles tendon of Gt (ROSA)26sor transgenic mouse were consistent with the aforementioned findings (Figure S16, Supporting Information). H&E and SOFG staining revealed well‐developed HO in the tendons of mouse in the HO group, which was reduced in the EN6‐treated mouse (Figure S16, Supporting Information). Additionally, immunostaining showed a decrease in the green fluorescent signal in the Achilles tendon of the HO+EN6 group suggesting that autophagosomes were hydrolyzed after binding to lysosomes and autophagic flux was restored (Figure 9G,H). Taken together, these results confirm that treatment with EN6 restores autophagic flux, which had been blocked in injured tendons and tendon HO. Therefore, EN6 could be a potential therapeutic drug for the treatment of autophagy‐lysosomal dysfunction that drives tendon HO.

## 3 Discussion

Pathologic calcification is the process of depositing calcium phosphate salts in soft tissue that has been injured, inflamed, or necrotic.^[^ ^37^ ^]^ While calcium phosphate deposition is detected early in tendon injuries, the mechanism behind its formation remains unknown.^[^ ^12^ ^]^ Therefore, further research is needed to clarify this relationship. Previous studies indicate that autophagy activation reduces the formation of tendon calcification.^[^ ^38^ ^]^ However, the impact of autophagy dysfunction on tendon calcification is unclear. Therefore, we employed a trauma‐induced tendon HO model to investigate the involvement of autophagy in tendon calcification for this study. First, we found through in vivo study that blocked autophagic flux is related with early tendon calcification. Using autophagy inhibitors, we found insufficient lysosomal degradation ability may be the main cause of impaired autophagic flux. Then we examined through in vivo experiments of transgenic mice and verified disruption of the acidic environment of lysosomes during development of HO. Second, we established an in vitro model using IL‐1β and TNF‐α to mimic the in vivo inflammation microenvironment of early HO. The results echoed the in vivo study that acidic dysfunction of lysosome led to HO. Using various blocking experiments in vitro, we found reduced activity of the V‐ATP6V1A subunit is the major cause of lysosome dysfunction, which can be rescued using a potent and selective activator of V‐ATP6V1A, EN6. Finally, we attempted to restore the acidic function of lysosomes to treat autophagic flux obstruction and tendon HO in vivo. The results showed that increasing the acidity of lysosomes can restore the autophagic flux and reduce the occurrence of tendon HO. These results indicate that V‐ATP6V1A‐mediated lysosome dysfunction is the major cause of impaired autophagic flux in the early stages of tendon HO.

Previous studies suggest that tendon rupture leads to heightened expression levels of autophagy‐related proteins LC3B and p62/SQSTM1.^[^ ^39^ ^]^ Our investigation implemented immunofluorescence staining to detect the expression of autophagy carrier proteins LC3B and p62, which were upregulated in the injured mouse tendon tissue of the HO groups, but scarcely present in the sham groups. Additionally, calcified junctions were observed in the injured mouse tendon tissue in the HO group as early as one week after the operation, and ectopic bone was observed in the tendon at three weeks. To assess the effect of hindered autophagic flux on tendon HO, we pharmacologically obstructed autophagic flux in tendon tissue post‐tenotomy. After treatment with CQ, a significant increase in p62 protein was observed. Tendon calcification was earlier and more severe in the injured mouse tendon tissue in the HO+CQ group compared to the HO group. The results suggest that impeding autophagy flux accelerates ectopic bone formation in tendons. Therefore, impaired autophagy flux exacerbates HO formation. Next, we examined the factors impaired responsible for the autophagic flux. To assess the location and flux of autophagy, we employed autophagy‐transgenic mice that express a tandem mCherry‐EGFP‐LC3 fusion protein, resulting mCherry (red fluorescent signal) and EGFP (green fluorescent signal) fluorescence in autophagosomes. Loss of this signal when autophagosomes bind to lysosomes at acidic pH due to the sensitivity of EGFP fluorescence to acidity. In tendon tissues of sham group mice, autophagy levels were observed to be low with a majority of autophagosomes expressing mCherry fluorescence. Conversely, enhanced autophagy levels were observed in tendon tissues of HO group mice; with a majority of autophagosomes expressing both mCherry and EGFP fluorescence, thereby confirming the accumulation of autophagosomes in the injured tendons. To determine the cause of autophagosome accumulation, we analyzed the co‐localization of autophagosome with lysosome‐anchored Ras‐related protein 7 (Rab7), a GTPase. We observed prominent co‐localization of EGFP signal with Rab7 in the tendon tissues of HO group mice, while the distribution of EGFP signal was negligible in the tendon tissues of sham group mice. The experimental findings indicate that autophagosomes are can bind to lysosomes for the creation of autophagic lysosomes. Lysosomal acidic dysfunction is determined as the primary cause of the disrupted autophagic flux. After tendon trauma in mice, although RAPA was able to drive the autophagic process by promoting autophagiogenesis and autophagosome‐lysosome binding,^[^ ^29^ ^]^ it was unable to correct the lysosomal degradation dysfunction and instead accelerated the accumulation of autophagosomes and their secretion. Therefore, RAPA treatment did not mitigate the obstruction of autophagy flux and the progression of HO. Furthermore, in vitro, we found that the fibroblast lysosomal acidic dysfunction induces the accumulation of autophagosome. RAPA promotes the generation of autophagosomes and their binding to lysosomes, but could not rescue the lysosomal acidic dysfunction. Although CQ and RAPA are two different drugs, they both have no effects on the rescue of lysosomal acidic dysfunction, and cause autophagosome accumulation and secretion, eventually promoting the calcification process. Fortunately, EN6 treatment restored the lysosomal acidification function, rescued the blocked autophagic pathway, and effectively reversed the progression of tendon HO. In conclusion, this study illustrates the role of the autophagy‐lysosomal pathway in the development of HO, which has important clinical implications for HO treatment.

Autophagy is a ubiquitous cellular mechanism for the removal and recycling of damaged proteins and organelles.^[^ ^40^ ^]^ At the basal level, this mechanism plays a key role in cellular quality control and homeostasis in vivo. Recent evidence suggests that autophagy plays an important role in mineralization. For example, selective blockade of autophagic flux in VSMC can accelerate the onset of vascular calcification.^[^ ^41^ ^]^ In the authors' previous study, impaired fusion of autophagosome with lysosome, caused by histone deacetylase 6‐mediated microtubule instability in chondrocytes, initiated cartilage calcification in osteoarthritis.^[^ ^11^ ^]^ However, the pathological calcification of osteoarthritic cartilage only involves mineral deposition in the extracellular matrix and does not involve cellular osteogenic process,^[^ ^42^, ^43^ ^]^ while HO forms bone‐like tissue.^[^ ^6^ ^]^ Although both diseases are caused by blocked autophagic flow, the mechanisms are different. Our previous findings suggest that microtubule destabilization in osteoarthritic chondrocytes results in autophagosomes secretion because of the inability of autophagosomes to bind with lysosomes, which induce the pathological calcification. In this study, abnormal lysosomal acidic function in fibroblasts caused blocked autophagic flow and led to HO. However, we still do not know how tendon HO disease causes abnormal lysosomal acidic function, and the mechanism of microtubule destabilization in osteoarthritic pathologic calcification has not yet been clarified. We hypothesized that this may be due to the different causative factors of these two diseases. Pathological calcification of osteoarthritic cartilage mainly results from long‐term exposure of abnormal forces.^[^ ^42^, ^44^ ^]^ HO described in this study is triggered by localized trauma‐caused acute inflammatory microenvironment. In addition, autophagy may mediate the intracellular concentration and distribution of calcium ions and phosphate.^[^ ^14^ ^]^ Mitochondria, endoplasmic reticulum and other organelles have been reported to be associated with calcium and phosphorus processing, and calcium and phosphorus may also be sequestered by autophagosomes.^[^ ^45^ ^]^ it is possible that, possible that, precursors of calcification such as Ca and Pi are initially formed or processed within a subcellular compartment such as endosomes, multi‐vesicular of Ca^+^ and Pi homeostasis in the cellular energy landscape.^[^ ^45^ ^]^ In addition, elevated cytoplasmic calcium concentrations (e.g., at the bottom in alginate treatment) induce autophagosome formation via a TOR‐dependent and independent pathway.^[^ ^46^ ^]^ Interestingly, these autophagosomes can be released into the stroma in the form of extracellular vesicles.^[^ ^47^ ^]^ Previous studies have shown that blocking the autophagic flux promotes more extensive calcification by facilitating the release of extracellular vesicles. In atherosclerosis, for example, VSMCs and macrophages are the major source of these calcified extracellular vesicles, which are released into the collagen‐rich matrix of the intima, promoting atherosclerotic calcification and leading directly to the formation of calcified plaques.^[^ ^48^, ^49^ ^]^

Although previous studies have confirmed that impaired autophagic flux mediates the formation of pathological extracellular matrix calcification through the release of extracellular vesicles, the cause of autophagic dysfunction remains unclear. Lysosomes have been reported to play a central role in the degradation of autophagosomes. For example, autophagosomes from primary osteoblasts of calcified mouse are filled with calcified hydroxyapatite.^[^ ^50^ ^]^ Acid lysosomes may play an important role in the lysis of such hydroxyapatite when fused to these autophagosomes.^[^ ^50^ ^]^ This supports the focus on lysosomal function in our study. In the present study, we found that autophagic flux was impaired in injured tendon tissue, but this did not affect fusion between autophagosome and lysosome. Further studies using mCherry‐EGFP‐LC3 transgenic mice model revealed that dysfunctional autolysosome degradation resulted in impaired autophagic flux. In the early localized trauma‐caused acute inflammatory microenvironment of tendon HO, we observed a significant increase in the expression of IL‐1β and TNF‐α factors (Figure S14, Supporting Information), which is consistent with the results of previous studies.^[^ ^51^, ^52^ ^]^ To establish an in vitro model of pathological calcification fibroblast, we applied IL‐1β and TNF‐α in vitro to mimic the in vivo inflammatory microenvironment of early HO, and found impaired lysosomal acidic function of fibroblasts, which resulted in blocked autophagic flow, and promoted mineralization. This suggested that the disruption of the acidic environment of the lysosome may be caused by inflammatory microenvironment. Further research is needed to determine whether regulating this inflammatory condition in vivo can rescue the acidity abnormality of lysosomes. In conclusion, we propose that lysosome acidic function play an important role in mediating pathological mineralization.

Lysosomes are single membrane‐bound organelles that contain acidic hydrolases responsible for degrading cellular cargo and maintaining cellular homeostasis.^[^ ^32^ ^]^ Lysosomes are characterized by a low internal pH of approximately 4.5–5.5. The acidic pH of lysosomes is mainly produced by the vesicular ATPase (V‐ATPase). The maintenance of a high acidic pH is essential for the regulation of many lysosomal functions. The wide range of pH optima suggests that the increase in intraluminal pH that accompanies substrate introduction during fusion with autophagosome or endosomes and the gradual reacidification of the lumen of the lysosomal compartment may coordinate the hydrolase activation sequence that is most effective for degrading and digesting complex substrates (e.g., mitochondria) or minimizing the production of amyloid or other potentially harmful digestion products. In the present study, we further investigated the mechanism of autolysosome degradation dysfunction in injured tendon tissue by analyzing lysosomal acidification function. We found abnormal lysosomal acidification function and inhibited maturation of lysosomal protease CTSD in injured tendon tissues. Therefore, based on the decreased acidity of lysosomes and the lack of CTSD maturation, we suggest that V‐ATPase is a potential target for HO treatment.

Based on these findings, we sought to explore therapeutic approaches to this pathological mineralization. V‐ATPase is a multimeric protein complex that acts as an ATP‐dependent proton pump and is present and active in almost all eukaryotic cells.^[^ ^32^ ^]^ V‐ATPase uses the energy from ATP hydrolysis to actively transport H‐ions into the lysosome, thereby making the lumen more acidic. In the present study, we found that reduced activity of the V‐ATP6V1A subunit is the major cause of V‐ATPase inactivation. Therefore, a potent and selective activator of V‐ATP6V1A, EN6,^[^ ^33^ ^]^ a small molecule in vivo activator of autophagy that covalently targets the ATP6V1A subunit of V‐ATPase to regulate lysosomal acidification, was used as an autophagy regulator in animal studies. EN6 was found to effectively promote autophagosome degradation and activate autophagy to prevent the progression of tendon HO. To our knowledge, this is the first study to use lysosomal intervention to activate autophagy to protect injured tendons from calcification. Such a strategy is promising for future translational applications.

**Figure 10:**
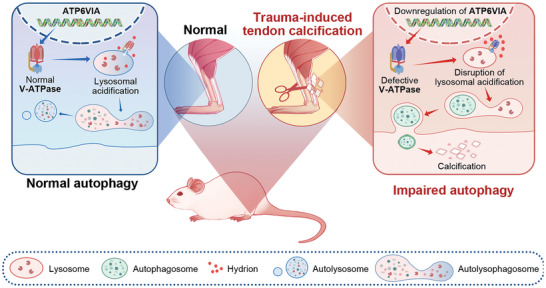
Schematic diagram depicting the involvement of the autophagy‐lysosome system in the formation of early HO in tendons. In the early stages of HO development, V‐ATPase dysfunction can lead to insufficient lysosomal acidification in damaged tendon tissue, which in turn hinders autophagy flux and accelerates tendon calcification. It is hypothesized that the accumulation of autophagosomes in damaged tendon tissue can facilitate the release of extracellular vesicles into the extracellular matrix, contributing to the formation of HO.

In conclusion, our current study suggests that autophagy‐lysosomal dysfunction is the major cause of impaired autophagic flux in the early stages of tendon HO. In the progression of tendon HO, we found that autophagic flux was impaired in injured tendon tissue, and further accumulation of autophagosome may exacerbate the progression of tendon calcification. We identified reduced lysosomal acidification capacity as a major cause of autophagosome and autolysosome accumulation. Our findings suggest a novel pathophysiological mechanism that may be exploited for the treatment of HO (**Figure** **10**).

## 4 Experimental Section

### Mice

Male C57BL/6J mice were provided by the Labo‐ratory Animal Center of the Fourth Military Medical University. R26‐e(CAG‐LSL‐mCherry‐EGFP‐LC3‐pA)1 Rosa26KI (referred to as Rosa26^LSL/+^) mice and Dppa3‐IRES‐Cre mice were established by Shanghai Biomodel Organism Science & Technology Development. Establishment of a Rosa26^LSL/+^: Cre mouse (Figure S4).

1. Preparation of Rosa26^LSL/+^ mouse strain and Dppa3‐IRES‐Cre mouse strain.
2. Raise each strain to 8 weeks of age to obtain adult Rosa26^LSL/+^ and Dppa3‐IRES‐Cre mice respectively.
3. Cross the adult Rosa26^LSL/+^ and Dppa3‐IRES‐Cre mice.

Establish Rosa26^LSL/+^ female / and Dppa3‐IRES‐Cre male or vice versa. There is no significant difference between their offspring. Typically, female mice give birth to 8–10 pups 19–21 days after mating. The offspring were genotyped by polymerase chain reaction (PCR) with genomic DNA (gDNA) as previously described using the primers listed in (Table S1, Supporting Information).

### Animal Model

Male C57BL/6J, WT (C57BL/6J genetic background, male) mice and Rosa26^LSL/+^: Cre (C57BL/6J genetic background, male) mice aged 6–8 weeks and weighing 22–25 g were employed in this study. To induce HO formation, male mice were subjected to Achilles tenotomy. Briefly, mice were anesthetized with 1% pentobarbital sodium. The skin was incised to expose the Achilles tendon. The Achilles tendon was clamped 20 times with hemostatic forceps and then cut with scissors. Finally, the skin was closed with sutures. Sham mice were subjected to the same procedures except Achilles tenotomy.

To assess the effect of CQ, RAPA, and EN6 on HO. Each mouse was laid sidewise after deep anesthesia with 1% intraperitoneal sodium pentobarbital. According to the manufacturer's instructions of EN6 (Cat# HY‐128892, MCE), RAPA (Cat# HY‐10219, MCE) and CQ (Cat# HY‐P2333A, MCE), 50 mg kg^−1^ EN6, 50 mg kg^−1^ RAPA, and 50 mg kg^−1^ CQ was injected locally into the HO region of the injection groups every other day.

All animals were maintained and handled under the approval of the Laboratory Animal Welfare and Ethics Committee of the Fourth Military Medical University (ethics approval number: 20230001, Xian, China). All mice were bred and maintained under SPF conditions with a 12‐hour dark/light cycle, regular chow diet, 24°C temperature, and 60% humidity.

### Micro‐Computed Tomography

The CT value of soft tissues in mice is about 300 Hu, while the CT value of hard tissues (e.g., bone) is more than 1000 Hu. The samples were examined via micro‐CT system (Inveon, Siemens, Germany), and regarded the region with the CT values exceeding 1000 Hu as ectopic ossification. Subsequently, the degree of HO was valued through analyses including bone mineral density (BMD), bone volume/tissue volume fraction (BV/TV), and trabecular thickness (Tb.Th) using GE Healthcare software.^[^ ^5^ ^]^

### Histological and Immunohistochemical Staining

Specimens were fixed in 4% paraformaldehyde, dehydrated in an ascending ethanol series, embedded in paraffin, deparaffinized, and rehydrated in xylene and a descending ethanol series. Hematoxylin and eosin (H&E), Safranin O/fast green (SOFG), and immunohistochemical staining of 5‐µm thick sections were performed according to the supplier's instructions, followed by light microscopy and observation. Digitized images of stained sections were analyzed using ImageJ software (NIH, Bethesda, MN, USA).

### Immunofluorescence Staining

To prepare the samples, fresh specimens were fixed in a 4% paraformaldehyde solution for 24 h, decalcified using a 0.5 m solution of EDTA for 2 weeks, and dehydrated in 30% sucrose solution for 24 h. After the preparation of samples, we embedded them in OCT (Thermo) and sectioned them at 5 µm intervals using a cryostat (Leica, Wetzlar, Germany). To perform immunofluorescence, frozen sections were blocked with goat serum for 30 min at 37 °C. Then, the sections were incubated overnight at 4 °C with primary antibodies such as LC3B (Cell Signaling, catalogue number: E5Q2K), p62 (Santa Cruz Biotechnology, catalogue number: sc‐376362), LAMP1 (Novus Biologicals, catalogue number: NBP1‐77461), RAB7 (Abcam, catalogue number: Ab137029), and V‐ATP6V1A (Abcam, catalogue number: ab199326). α‐SMA (Affinity, catalogue number: #AF1032), Collagen I (Affinity, catalogue number: #AF7001). For detection, secondary antibodies were used conjugated with fluorescent dyes (Beyotime/Abcam) at a 1:500 dilution for 1 h at 37 °C. The cell nuclei were stained using 4′,6‐diamidino‐2‐phenylindole (DAPI; ab228549; Abcam) at room temperature for 15 minutes. For Alizarin Red S staining, the frozen sections were stained with Alizarin Red S (40 mm, pH 4.2; Millipore Sigma, Burlington, USA) for 20 min. The nuclei were counterstained with 4′,6‐diamidino‐2‐phenylindole. The stained sections were imaged using laser scanning confocal microscopy (CLSM) (Nikon AIP, Nikon Corporation, Minato‐ku, Tokyo, Japan). The relative fluorescence intensity was measured using the ImageJ software (National Institutes of Health, Bethesda, MD, USA).

### Western Blot Analysis

Tissues were collected from the Achilles tendon tissues of mice after surgery, ground with glass pestles in RIPA buffer on ice, and subsequently sonicated to extract tissue proteins. The samples were divided by 10% sodium dodecyl sulfate‐polyacrylamide gelelectrophoresis (SDS‐PAGE) after which they were transferred to polyvinylidene difluoride membranes (IPVH00010, Millipore). Primary antibodies were used for immunoblotting against the following proteins: anti‐CTSD (ET1608‐49, HUABIO, China). After electrophoresis, the polyvinylidene difluoride membranes were incubated with secondary goat anti‐mouse IgG horseradish peroxidase‐conjugated antibody (ab150113, Abcam, USA). Glyceraldehyde 3‐phosphate dehydrogenase anti‐GAPDH (ET1702‐66, HUABIO, China) was used as internal control. The stained bands were quantified using image J software.

### Quantitative Real‐Time Polymerase Chain Reaction(qRT‐PCR)

To extract total RNA, TriPure Isolation Reagent (11667165001) from Roche, Indianapolis, IN, USA was used following the manufacturer's instructions on mouse Achilles tendon tissue. Complementary DNA was synthesized, and real‐time polymerase chain reaction (RT‐PCR) was carried out. Quantitative RT‐PCR was conducted using FastStart Universal SYBP Green Master reagent (Roche) and primer pairs on an ABI Step One‐Plus instrument (Applied Biosystems, Thermo Fisher Scientific). Gene expression levels were estimated using the 2−ΔΔCt method with glyceraldehyde‐3‐phosphate dehydrogenase (GAPDH) gene expression level as an internal control. The primer sequences are listed in Table S2 (Supporting Information).

### Scanning Electron Microscopy (SEM)and X‐Ray Energy Spectrometer (EDS)

Fresh Achilles tendon tissue was fixed in 2.5% glutaraldehyde and dehydrated through an ascending ethanol series (30%, 50%, 70%, 80%, 90%, and 100% ethanol). The specimen was then cover slipped with hexamethyldisilane (Electron Microscopy Sciences, Hatfield, PA, USA) and slowly air dried. Finally, it was sputtered with gold and examined with a field emission scanning electron microscope (FE‐SEM, S‐4800, Hitachi, Tokyo, Japan) at 5 kV. EDS (Element EDS System, Ametek, Berwyn, PA, USA) was used to characterize the mineral elemental composition in tendon tissue.

### Atomic Force Microscopic (AFM)

Atomic force microscopy (AFM) was used to analyze 7 µm paraffin sections. The instrument used was the Keysight 5500 from Keysight Technologies, Santa Rosa, CA, USA. Once the hydrated sections dried naturally, a silicon probe (PPP‐NCLR‐20, Nanosensors^TM^, Neuchatel, Switzerland) with a force constant of 42 N m^−1^ and a resonance frequency of 161 kHz was used to perform micromorphological imaging of mouse Achilles tendon tissue. Measurements were taken at six locations with each tissue sample and averaged.

### Statistical Analysis

The analyses was performed using GraphPad Prism 8.0 software (GraphPad Software, USA). All the data are presented as mean ± standard error of the mean deviation (SD). We used the Shapiro‐Wilk test and modified Leven tests to check the normality and homoscedasticity assumptions, respectively, of the data sets. The Student's *t*‐test, one‐ or two‐way analysis of variance (ANOVA) followed by the Holm‐Šidák multiple comparison test were used to evaluate differences among groups. The level of statistical significance was set at α = 0.05 for all the tests.

## Conflict of Interest

The authors declare no conflict of interest.

## Supporting information

Supporting Information

## Data Availability

The data that support the findings of this study are available from the corresponding author upon reasonable request.
