# Supplementary material for: Exploring the Link Between Autophagy‐Lysosomal Dysfunction and Early Heterotopic Ossification in Tendons
Source: Adv Sci (Weinh). 2024 May 13;11(28):2400790. doi: 10.1002/advs.202400790 (PMC11267276; doi:10.1002/advs.202400790)
Supplement: Supplementary file 1 — Supporting Information [file ADVS-11-2400790-s001.pdf]

ADVANCED SCIENCE
Open Access

Supporting Information

for *Adv. Sci.*, DOI 10.1002/advs.202400790

Exploring the Link Between Autophagy-Lysosomal Dysfunction and Early Heterotopic Ossification in Tendons

*Chang-He Gao, Qian-Qian Wan, Jan-Fei Yan, Yi-Na Zhu, Lei Tian, Jian-Hua Wei, Bin Feng, Li-Na Niu and Kai Jiao**

Supporting Information

Exploring the Link Between Autophagy-Lysosomal Dysfunction and Early Heterotopic Ossification in Tendons

Chang-He Gao#, Qian-Qian Wan#, Jan-Fei Yan#, Yi-Na Zhu, Lei Tian, Jian-Hua Wei, Bin Feng Li-Na Niu, and Kai Jiao**

Table of Contents

SI-1. Table S1. Primers for RT-qPCR [Supplementary Table 1]

SI-2. Table S2. Primers for RT-qPCR [Supplementary Table 2]

SI-3. Trauma -induces tendon calcification with progressive aggravation in mouse model [Figure S1]

SI-4. Trauma -induces tendon calcification with progressive aggravation in mouse model [Figure S2]

SI-5. Trauma -induces tendon calcification with progressive aggravation in mouse model [Figure S3]

SI-6. Impaired autophagic flux was found in injured tendons [Figure S4]

SI-7. Impaired autophagic flux was found in injured tendons [Figure S5]

SI-8. Impaired autophagic flux can exacerbate the progression of tendon calcification [Figure S6]

SI-9. Impaired autophagic flux can exacerbate the progression of tendon calcification [Figure S7]

SI-10. Tendon damage leads to impaired autophagic flux but does not affect the fusion of AP with LY [Figure S8]

SI-11. Tendon damage leads to impaired autophagic flux but does not affect the fusion of AP with LY [Figure S9]

SI-12. Tendon damage leads to impaired autophagic flux but does not affect the fusion of AP with LY [Figure S10]

SI-13. Damage of autophagy-lysosome system in the fibroblasts calcification model in vitro [Figure S11]
SI-14. Damage of autophagy-lysosome system in the fibroblasts calcification model in vitro [Figure S12]
SI-15. Damage of autophagy-lysosome system in the fibroblasts calcification model in vitro [Figure S13]
SI-16. Blocked autophagy flux facilitates release of extracellular vesicles to promote extracellular matrix calcification [Figure S14]
SI-17. Topical treatment to restore lysosomal acidification capacity can attenuate HO [Figure S15]
SI-18. Topical treatment to restore lysosomal acidification capacity can attenuate HO [Figure S16]

SI-1. Table S1. Primers for RT-qPCR [Supplementary Table 1]

Genes	Description	Primer sequences (5' →3')
Rosa26$^{LSL/+}$	P1	TCAGATTCTTTTATAGGGGACACA
	P2	TAAAGGCCACTCAATGCTCACTAA
	P3	GGTGTTGTCGGGGAAATCATCGTC
	P4	AGGAGCCTGCCAAGTAAC
Cre transgene mouse	P1	TGGGTTGGGTGTCTGTTTCATTGT
	P2	GATCCACCTGTCTCTGCCTTCC
	P3	GACCTTGCATTCCTTTGGCGAGAG

SI-2. Table S2. Primers for RT-qPCR [Supplementary Table 2]

Genes	Description	Primer sequences (5' to 3')
STX17	m- STX17-F	TCAAAGTGGCAGGAATTGCAG
	m- STX17-R	AATTTTCCACCTGTGAAGCCTAA
Snap29	h- Snap29-F	TTCGACGATGACGTGGAAGAG

	h- Snap29-R	GGTACTGCTGCCTGTCAATGG
Vamp8	h- Vamp8-F	GGGAGTGCCGGAAATGACC
	h- Vamp8-R	TGAAGTGTTCAGACGTGGCTT
LC3B	h- LC3B-F	CGCTTGCAGCTCAATGCTAAC
	h- LC3B-R	CTCGTACACTTCGGAGATGGG
P62/SQSTM1	h-SQSTM1-F	GAGGCACCCCGAAACATGG
	h-SQSTM1-R	ACTTATAGCGAGTTCCCACCA
V-ATP6V1D	m-ATP6V1D-F	GGTAACCGGTTCTGGAGGTG
	m-ATP6V1D-R	TTTCGACCAGTCTGTGCTCC
V-ATP6V1E	m-ATP6V1E-F	ATGGGGATCGCAAGATAAAGG
	m-ATP6V1E-R	CCACGGACTTCTGGCATCA
V-ATP6V1H	m-ATP6V1H-F	TATGGCTCCTGGCATTCAGTC
	m-ATP6V1H-R	GAGCATACTTGGCGAGTTTCT
V-ATP6V1B	m-ATP6V1B-F	GCTGTCATGGCAGAGGAGTT
	m-ATP6V1B-R	CGGGGTAGATTCGGTCATGG
V-ATP6V1A	m-ATP6V1A-F	CAATCACCCCTTGCTTACTGG
	m-ATP6V1A-R	TCTCACCGCAGCCGACATA
V-ATP6V1C	m-ATP6V1C-F	TTCCAGTATAACGAGGAGGAGATG
	m-ATP6V1C-R	CCGCACAAGTGGTCCAAAC
V-ATP6V0D	m-ATP6V0D-F	ATCGAGATAATCCGAAATACGC
	m-ATP6V0D-R	GAGTTGATGGTGATGATGAAAGC
V-ATP6AP1	m-ATP6AP1-F	TGAGCCACTCTTTGGTGCAA

m-ATP6AP1-R	TCGGGCAGACACTGGATAGA

SI-3. Trauma -induces tendon calcification with progressive aggravation in mouse model [Figure S1]

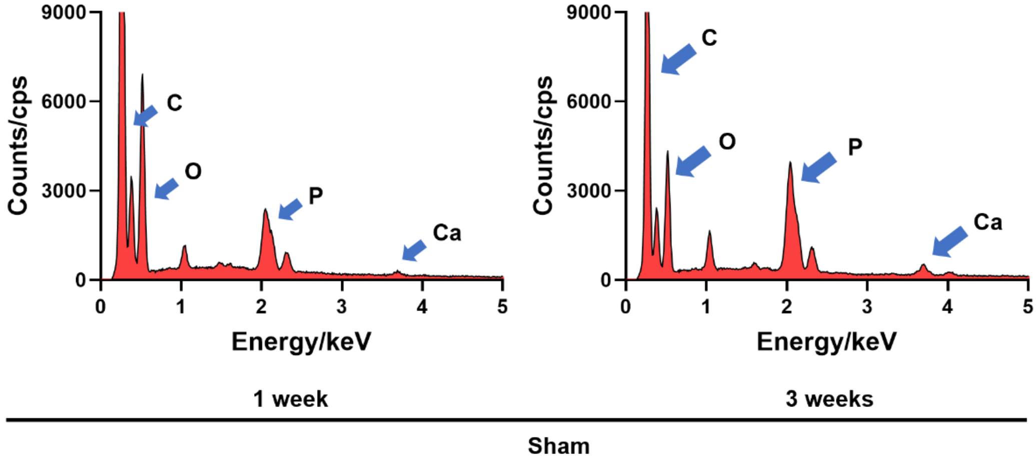

Figure S1. Trauma -induces tendon calcification with progressive aggravation in mouse model. Elemental analysis of the collagen fibers in 1-week sham group and 3-weeks sham group in Figure 1f.

SI-4. Trauma -induces tendon calcification with progressive aggravation in mouse model [Figure S2]

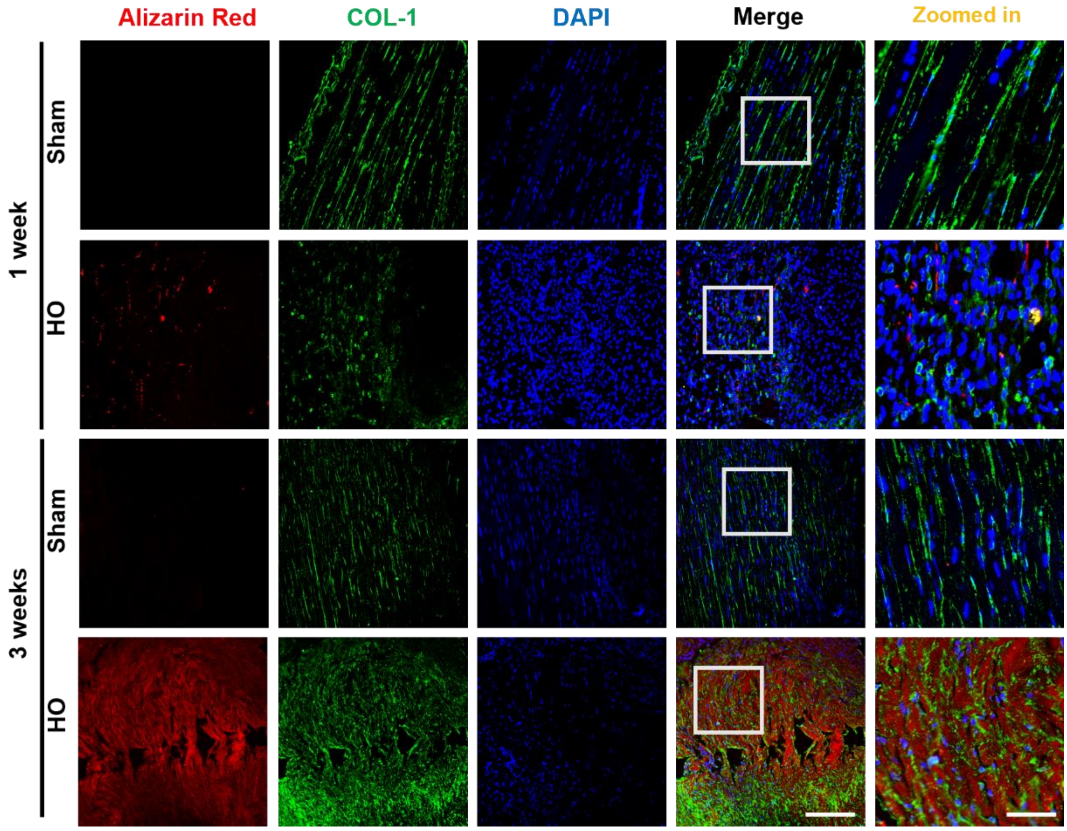

Figure S2. Trauma -induces tendon calcification with progressive aggravation in mouse model, related to Figure1. Representative local fluorescence images of the Achilles tendon from sham group and HO group mice at 1 week and 3 weeks after surgery (n=3). Alizarin Red S fluorescence indicates calcification within collagen. Type I collagen, green; DAPI, blue. Scale bar, 100 μm.

SI-5. Trauma -induces tendon calcification with progressive aggravation in mouse model [Figure S3]

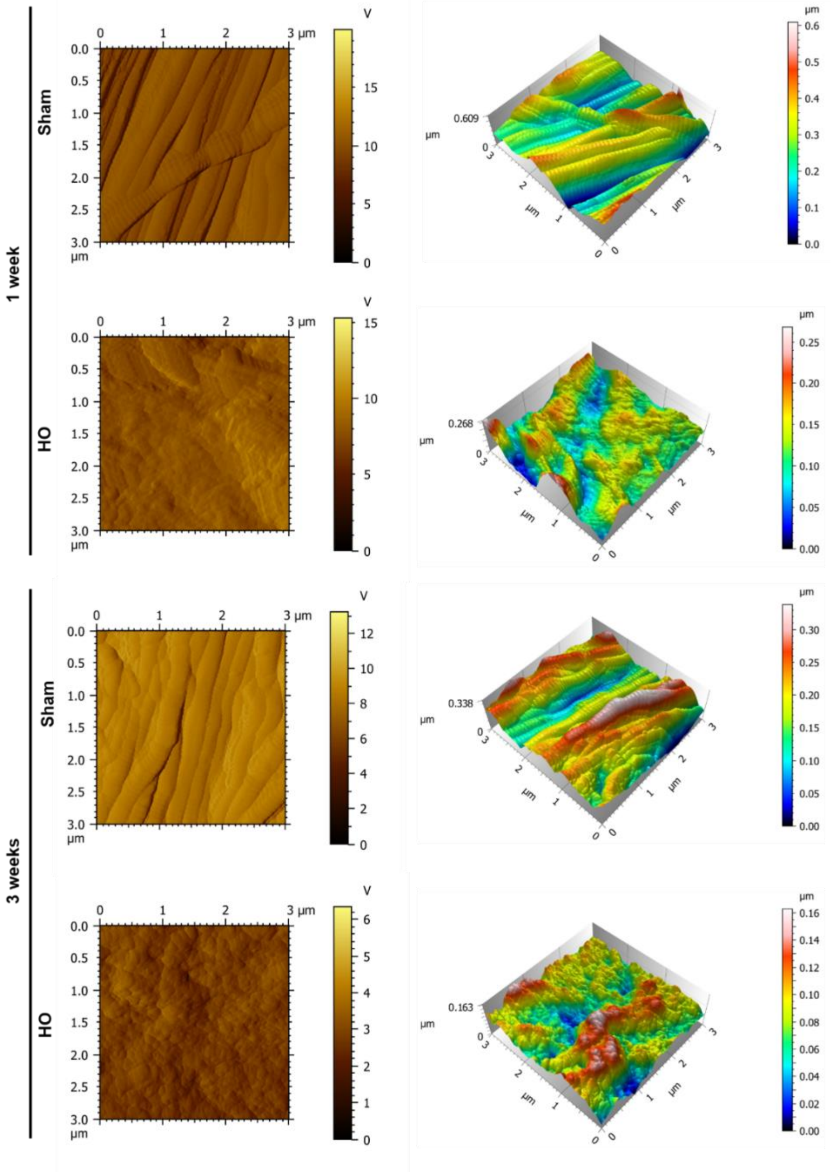

Figure S3. Trauma -induces tendon calcification with progressive aggravation in mouse model, related to Figure1. Atomic force scanning topography image and corresponding three-dimensional reconstruction image of the Achilles tendon from sham groups of and HO groups mice at 1 week and 3 weeks after surgery.

SI-6. Impaired autophagic flux was found in injured tendons [Figure S4]

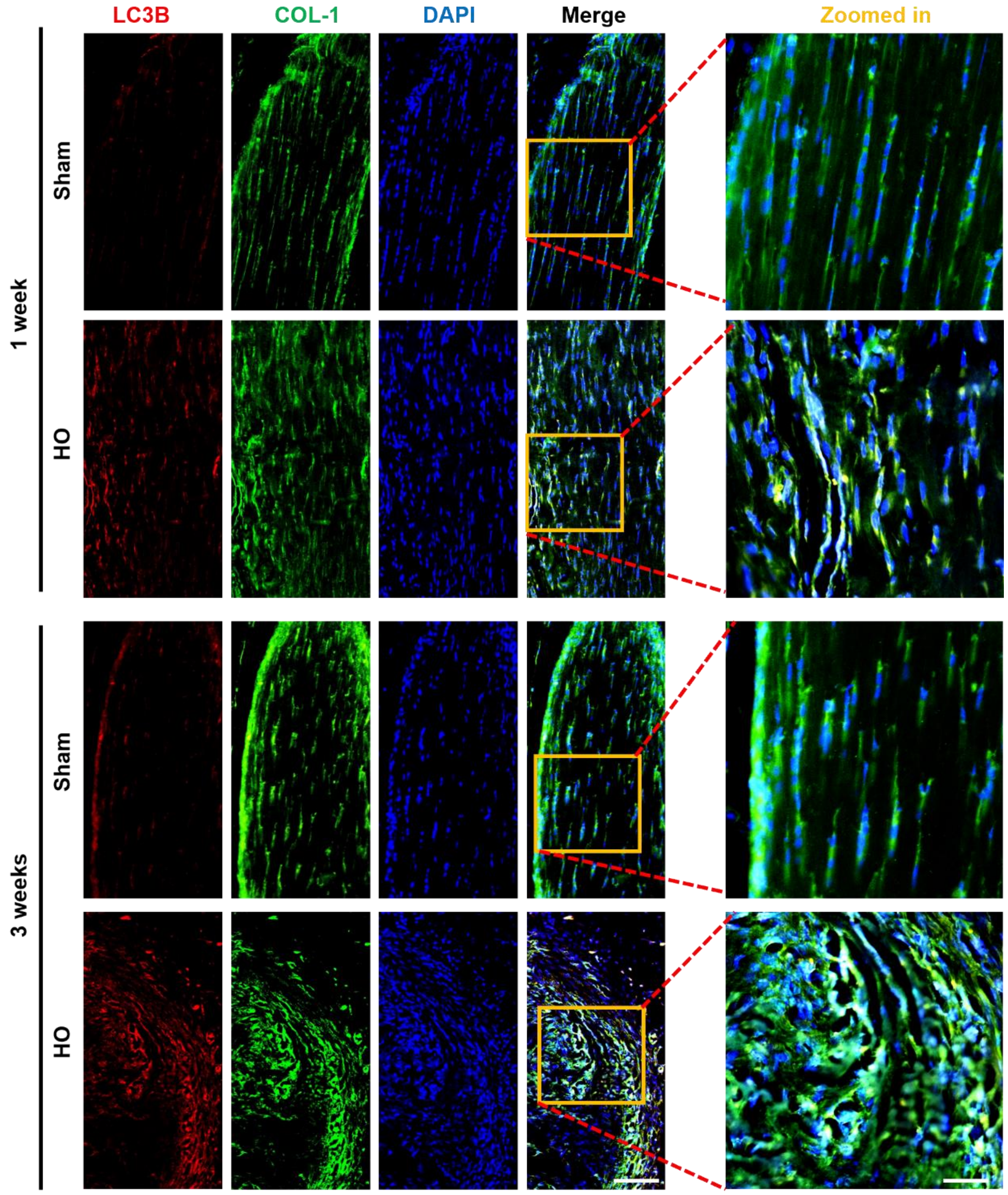

Figure S4. Impaired autophagic flux was found in injured tendons, related to Figure 2. Representative local fluorescence images of the Achilles tendon from sham group and HO group mice at 1 week and 3 weeks after surgery, co-labelled with the autophagosome and Type I collagen (n=3). Scale bar, 100 μm.

SI-7. Impaired autophagic flux was found in injured tendons [Figure S5]

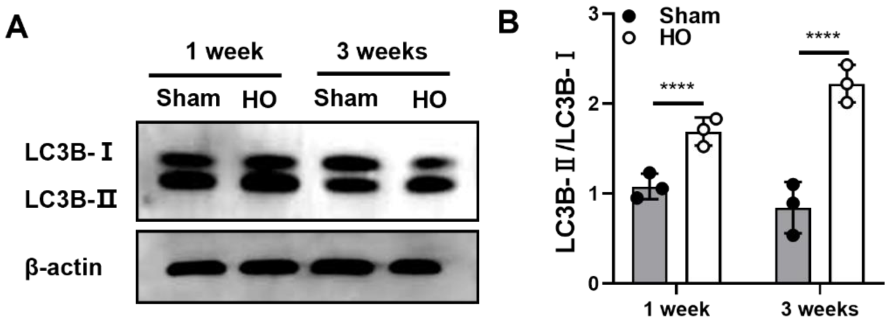

Figure S5. Impaired autophagic flux was found in injured tendons, related to Figure 2. (A) Western blot analysis of LC3B-II/LC3B-I protein in the Achilles tendon from sham groups and HO group at 1 week and 3 weeks after surgery. (B) The ratio of LC3B-II to LC3B-I was quantified in the bar graph (n=3). Statistical analyses are performed by two-way ANOVA with Holm-Šidák multiple comparison tests. ****P <0.0001.

SI-8. Impaired autophagic flux can exacerbate the progression of tendon calcification [Figure S6]

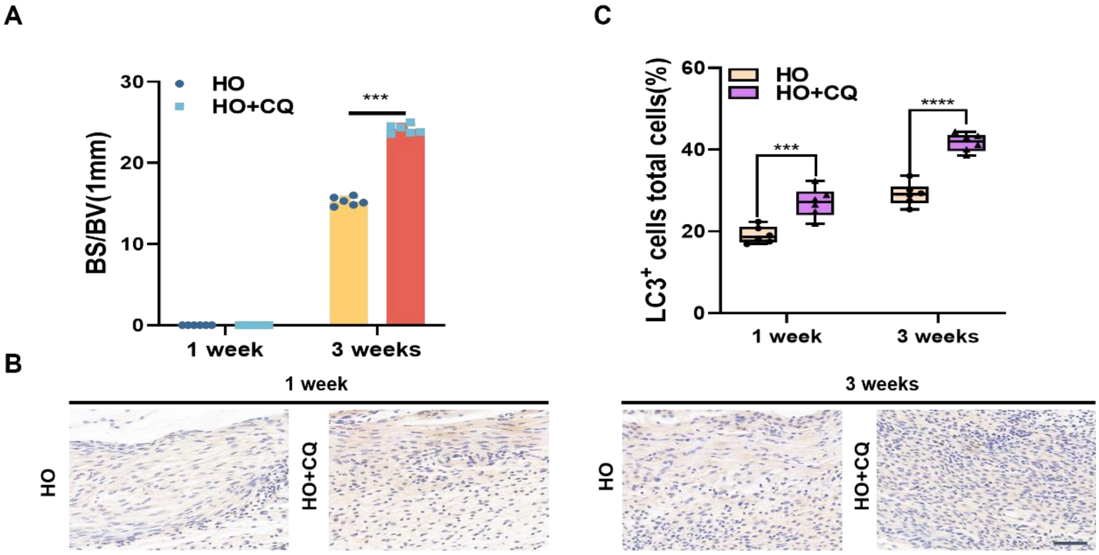

Figure S6. Impaired autophagic flux can exacerbate the progression of tendon calcification, related to Figure 3. (A) Quantitative analysis of trabecular thickness (BS/BV) in HO groups and HO+CQ groups mice at 1 and 3 weeks after surgery in Figure 3a. n=6 per group. (B and C) Representative immunohistochemical staining of the Achilles tendon from HO groups and HO+CQ groups mice to detect the number of LC3-positive cells. n=6 per group, Scale bar: 30 μm. Statistical analyses are performed by two-way ANOVA with Holm-Šidák multiple comparison tests. ****P*<0.001; *****P*<0.0001.

SI-9. Increased autophagy flux does not alleviate the progression of tendon calcification [Figure S7]

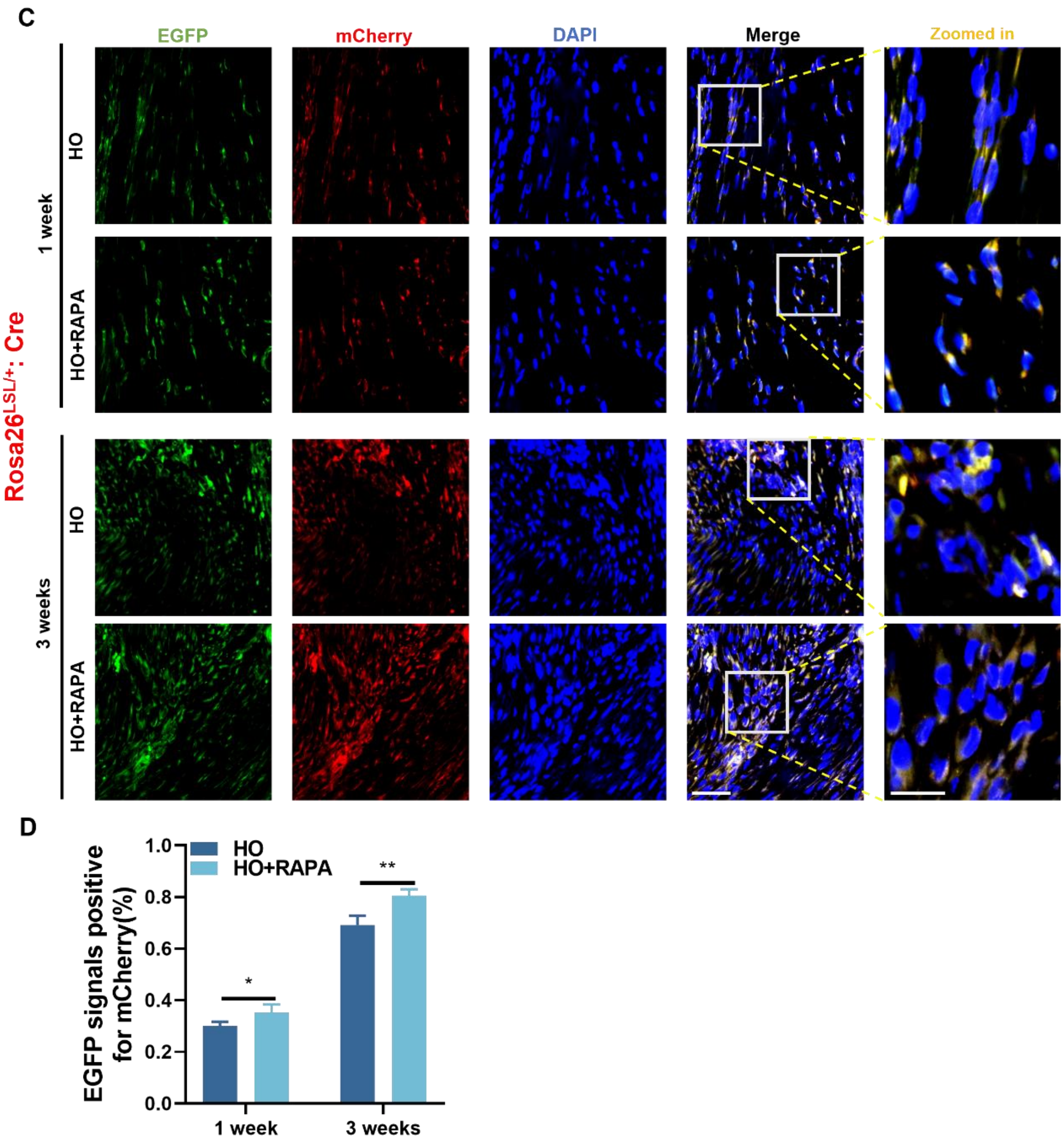

Figure S7. Increased autophagy flux does not alleviate the progression of tendon calcification, related to Figure 4. (C) Representative images of local fluorescence changes in the Achilles tendon from sham group and HO group at 1 week and 3 weeks after surgery. Scale bar, 50 µm. (D) Quantification of relative to EGFP fluorescence intensity on mCherry in a bar graph (n=3). Statistical analyses are performed by two-way ANOVA with Holm-Šidák multiple comparison tests. *P<0.05; ** P<0.01.

SI-10. Tendon damage leads to impaired autophagic flux but does not affect the fusion of AP with LY [Figure S8]

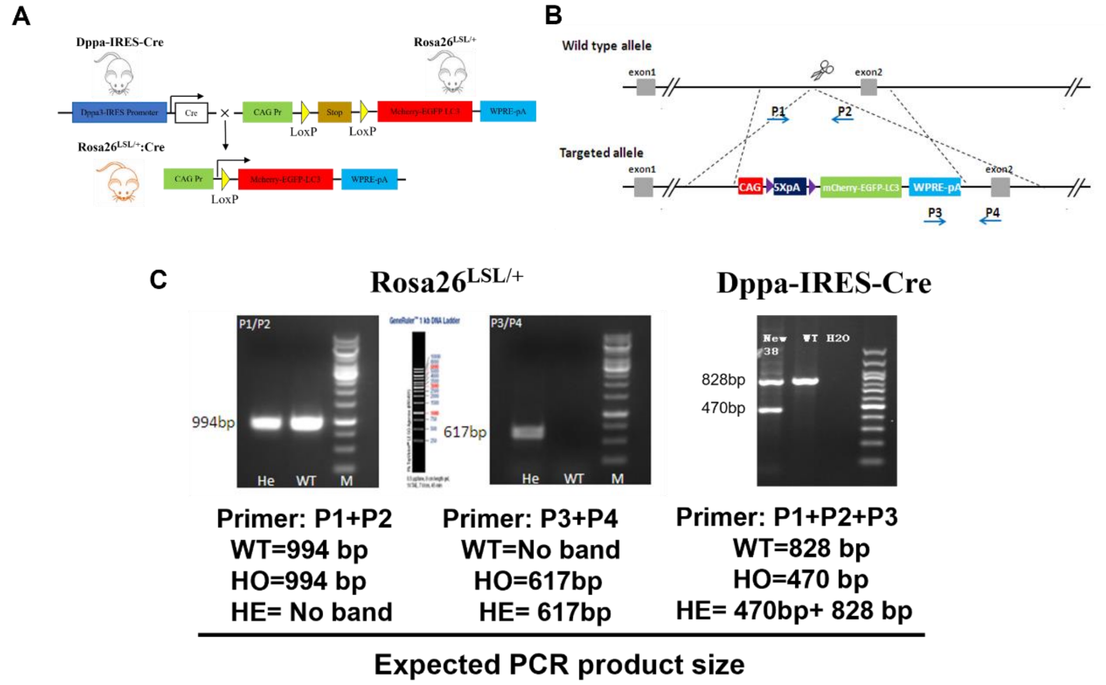

Figure S8. Tendon damage leads to impaired autophagic flux but does not affect the fusion of AP with LY, related to Figure 5. (A) Schematic of the Rosa26$^{LSL/+}$: Cre reporter mice. (B) Schematic representation of primer positions for PCR identification. (C) Sequencing of PCR identified products of Gt (ROSA)26sor transgenic mouse and target sequence comparison results.

SI-11. Tendon damage leads to impaired autophagic flux but does not affect the fusion of AP with LY [Figure S9]

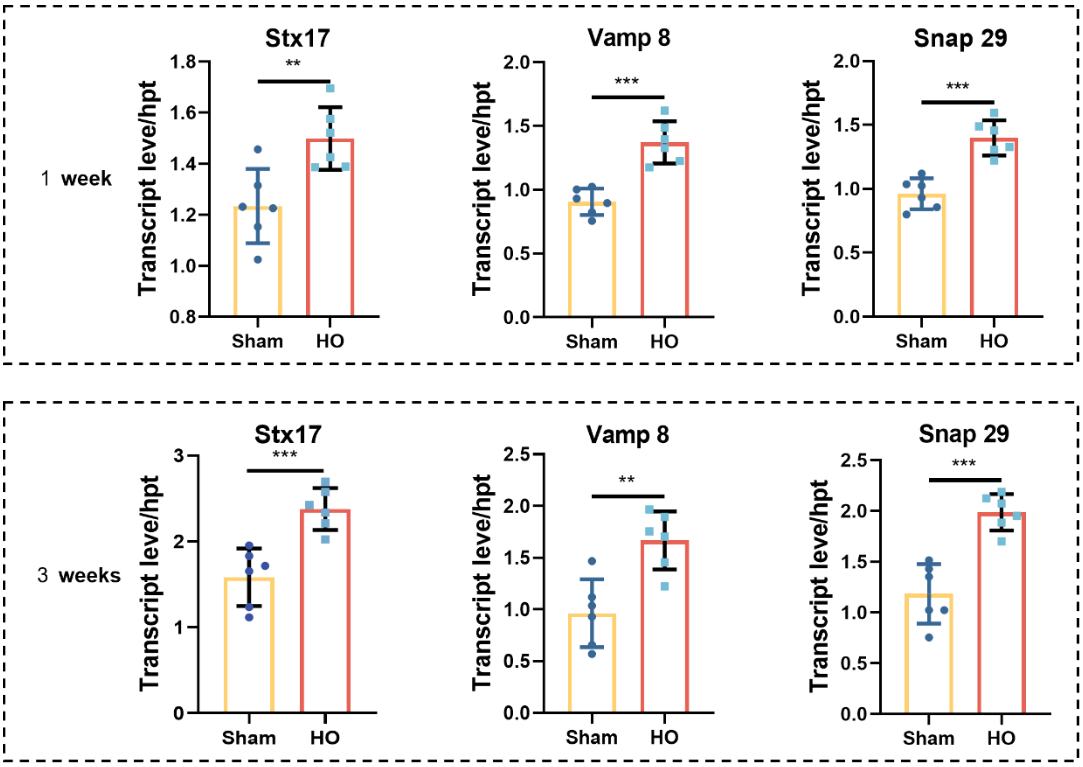

Figure S9. Tendon damage leads to impaired autophagic flux but does not affect the fusion of AP with LY, related to Figure 4. Quantitative real-time polymerase chain reaction analysis of the gene expression of the autophagosome-lysosome fusion-related factors in the Achilles tendon from sham groups and HO groups mice at 1 and 3 weeks after surgery. n=6 per group. Statistical analyses were performed using Student's t-test. $^{**}P<0.01$, $^{***}P<0.001$.

SI-12. Tendon damage leads to impaired autophagic flux but does not affect the fusion of AP with LY [Figure S10]

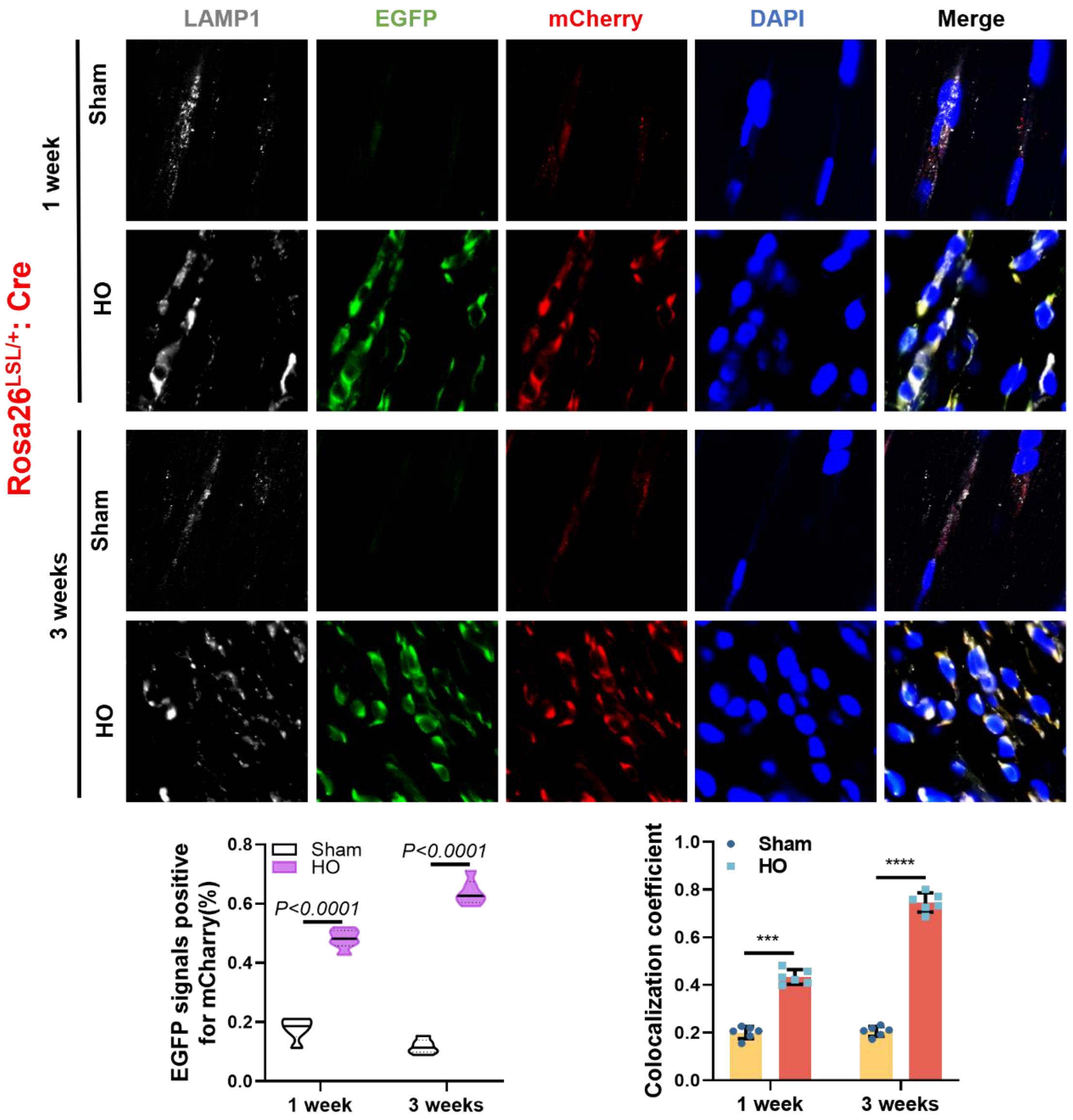

Figure S10. Tendon damage leads to impaired autophagic flux but does not affect the fusion of AP with LY, related to Figure 5. Representative local fluorescence images of the Achilles tendon from sham groups and HO groups mice at 1 and 3 weeks after surgery, co-labelled with the autophagosome and lysosomal membrane protein LAMP1(grey fluorescence indicator of lysosomes). DAPI, blue. n=6 per group, Scale bar: 25 µm, Scale bar, 100 µm. Statistical analyses are performed by two-way ANOVA with Holm-Šidák multiple comparison tests. ***P<0.001; ****P<0.0001.

SI-13. Damage of autophagy-lysosome system in the fibroblasts calcification model in vitro [Figure S11]

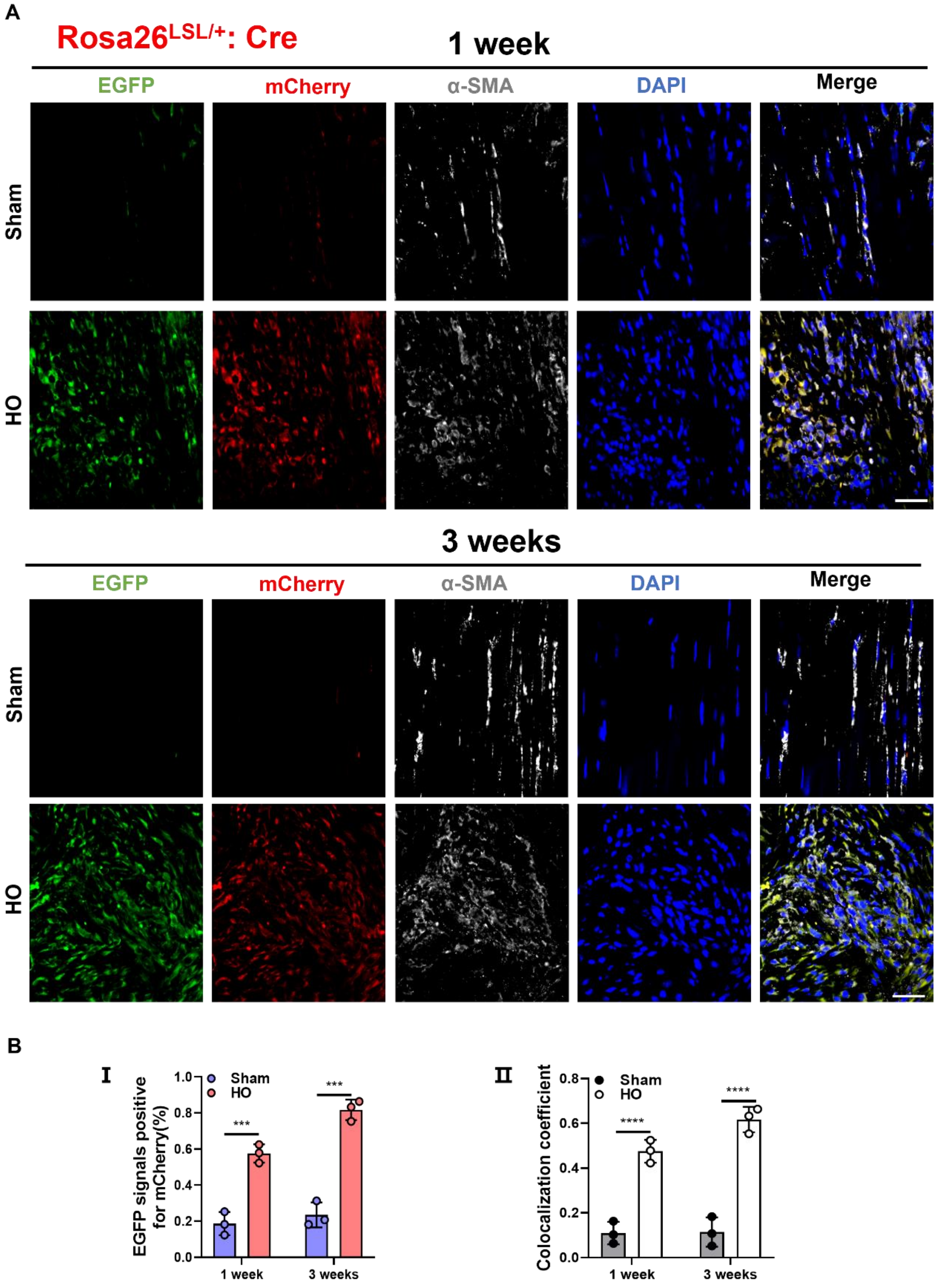

Figure S11. Damage of autophagy-lysosome system in the fibroblasts calcification model in vitro, related to Figure 7. Representative local fluorescence images of the Achilles tendon at 1 week and weeks after surgery from sham group and HO group using Gt (ROSA)26sor transgenic mouse, co-labelled (A) and quantified (B) with the autophagosome and Fibroblast-related markers α-SMA (n=3). Scale bar, 50 μm. Statistical analyses are performed by two-way ANOVA with Holm-Šidák multiple comparison tests. ***P<0.001. ****P<0.0001.

SI-14. Damage of autophagy-lysosome system in the fibroblasts calcification model in vitro [Figure S12]

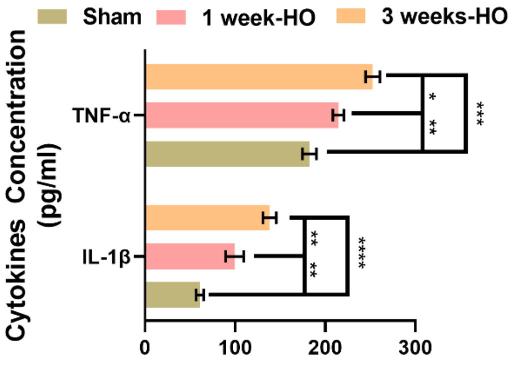

Figure S12. Damage of autophagy-lysosome system in the fibroblasts calcification model in vitro, related to Figure 7. ELISA results showing concentration of IL-1β and TNF-α, in the tendon tissue (n=3). Statistical analyses are performed by two-way ANOVA with Holm-Šidák multiple comparison tests. *P<0.05; **P<0.01; ***P<0.001; ****P<0.0001.

SI-15. Damage of autophagy-lysosome system in the fibroblasts calcification model in vitro [Figure S13]

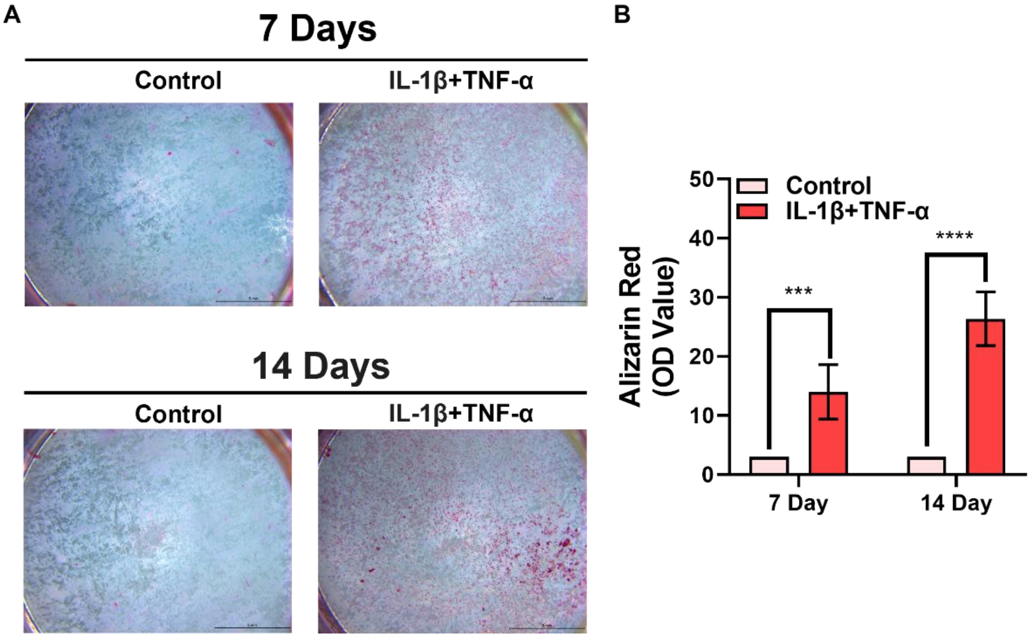

Figure S13. Damage of autophagy-lysosome system in the fibroblasts calcification model in vitro, related to Figure 7. (A) Alizarin red staining for calcification detection in tendon fibroblasts that were with IL-1β+TNF-α or/and EN6 or/and CQ or/and RAPA or/ and GW4869 for 7 and 14 days. (B) The bar graphs show the quantification of Alizarin red in the cells (n=3). Statistical analyses are performed by two-way ANOVA with Holm-Šidák multiple comparison tests. ***P<0.001. ****P<0.0001.

SI-16. Blocked autophagy flux facilitates release of extracellular vesicles to promote extracellular matrix calcification. [Figure S14]

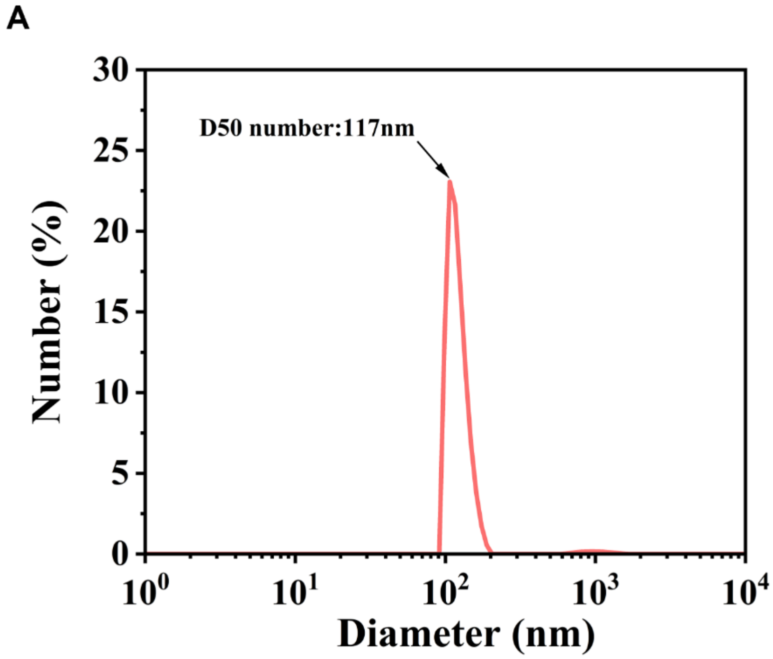

Figure S14. Blocked autophagy flux facilitates release of extracellular vesicles to promote extracellular matrix calcification, related to Figure 8. (A) Size distribution of exosomes analyzed by granulometric analysis.

SI-17. Topical treatment to restore lysosomal acidification capacity can attenuate HO [Figure S15]

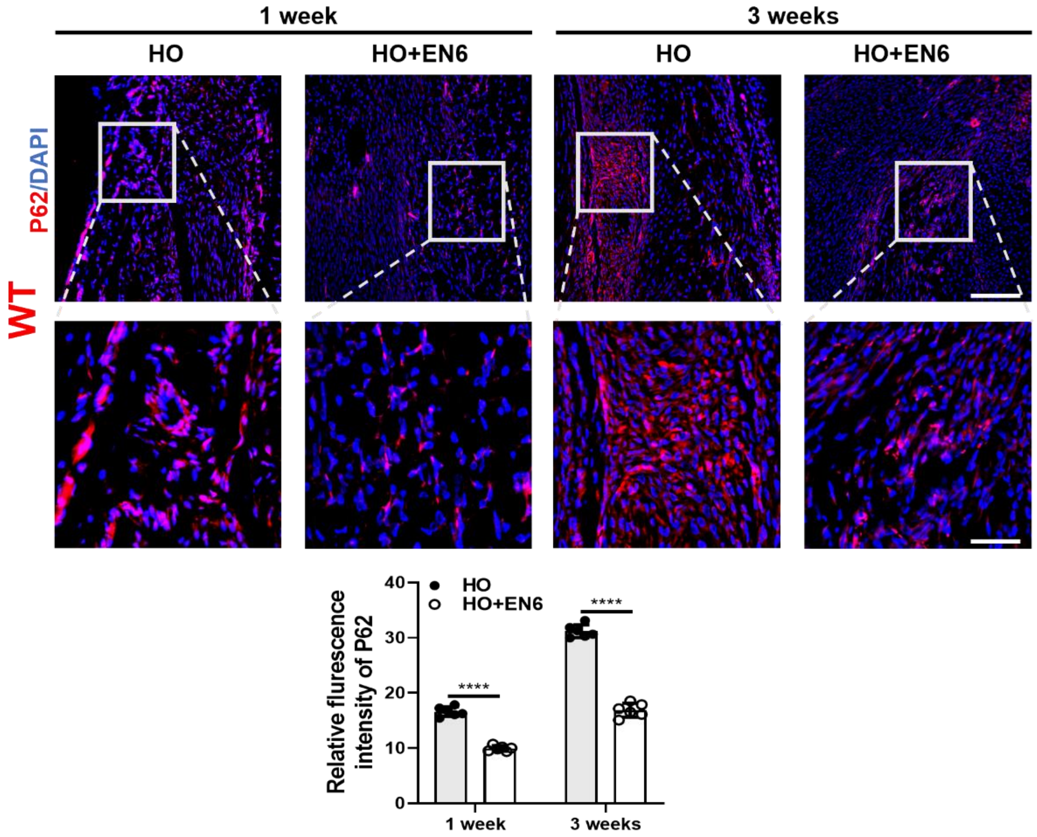

Figure S15. Topical treatment to restore lysosomal acidification capacity can attenuate HO, related to Figure 9. Representative confocal images of P62 (red fluorescence; indicator of autophagy) and DAPI (blue) immunostaining Achilles tendon in HO groups and HO+NE6 groups mice at 1 and 3 weeks after surgery. n=6 per group, Scale bar, 100 µm. Statistical analyses are performed by two-way ANOVA with Holm-Šidák multiple comparison tests. *****P*<0.0001.

SI-18. Topical treatment to restore lysosomal acidification capacity can attenuate HO [Figure S16]

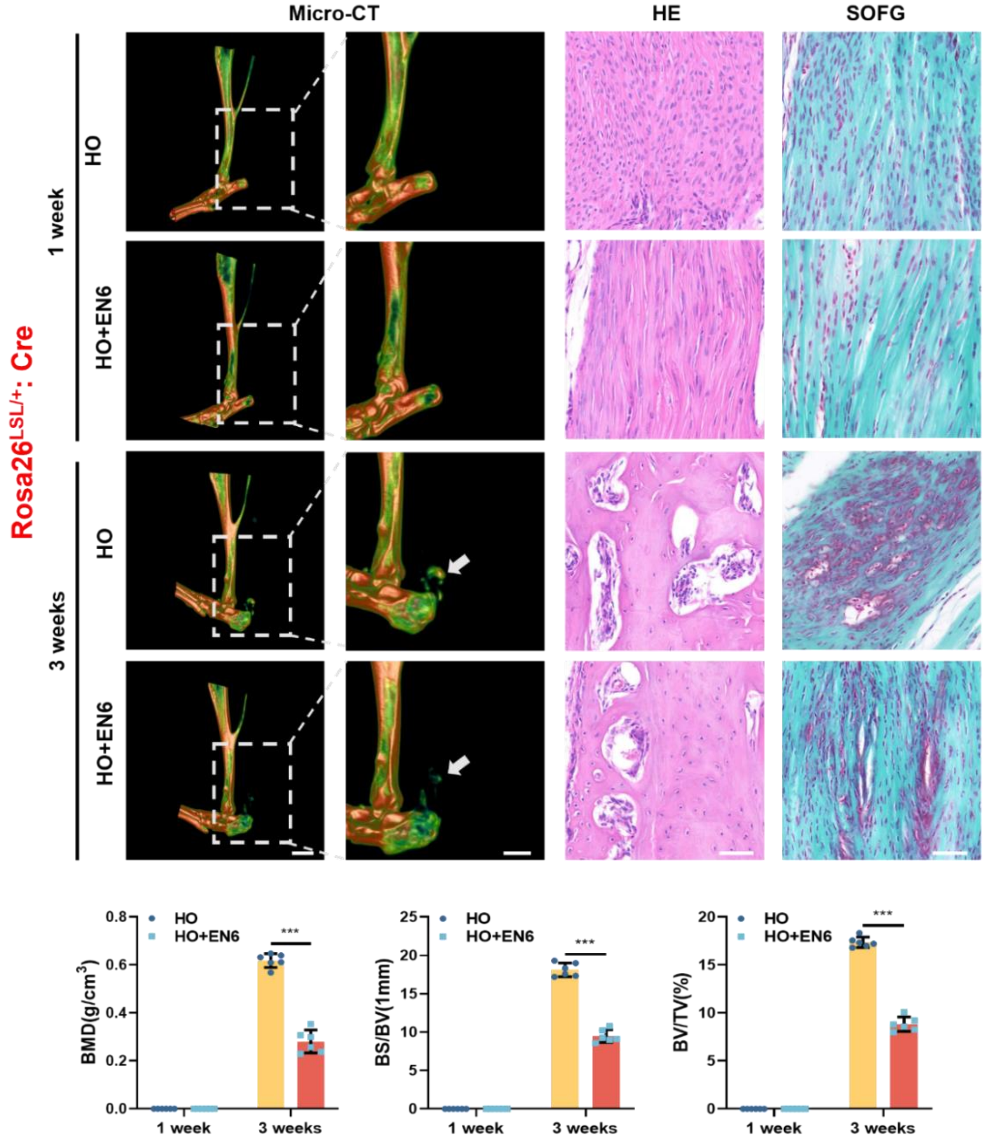

Figure S16. Topical treatment to restore lysosomal acidification capacity can attenuate HO, related to Figure 9. Representative micro-CT images of the Achilles tendon from HO groups of and HO+NE6 groups mice at 1 and 3 weeks after surgery (Arrows denote heterotopic calcification). Scale bar: 2 mm. Quantitative analysis of bone volume (TV/BV), bone mineral density (BMD) and trabecular thickness (BS/BV). Statistical analyses are performed by two-way ANOVA with Holm-Šidák multiple comparison tests. ***P<0.001.

Experimental Section

1. Cell isolation and culture

Repaired Achilles tendons of post-surgery mice were obtained, cut into pieces, and digested with 3 mg/ml type I collagenase (17100-017, Gibco) in the incubator for 3 h. The Tendon fibroblasts were migrated from the explant tissues, harvested with 0.25% of trypsin containing ethylenediaminetetra-acetic acid, and seeded into appropriate culture plates. Then, the tendon fibroblasts were grown to around 90% confluency, followed by the replacement of control medium or IL-1β and TNF-α medium. The control medium consisted of Dulbecco's modified Eagle's media (Gibco, Thermo Fisher Scientific, Waltham, MA, USA) supplemented with 10% fetal bovine serum (FBS) (PC-00001, PlanChemMed Biology Co.) and 1% penicillin-streptomycin solution (PC-86115, PlantChemMed Biology Co.). In IL-1β and TNF-α, when cell growth reached about 90% confluency, cells were washed with PBS three times and then grown in the IL-1β and TNF-α medium consisted of control medium supplemented with 10 ng/mL TNF-α, 5 ng/mL IL-1β, 10 nM dexamethasone, 100 μM l-ascorbic acid, 1.1 mM calcium chloride, and 10 mM β-glycerol phosphate (all from MilliporeSigma). The respective medium was replaced every 3 days.

2. LysoTracker assay

The lysosomal activity of the cells was measured using LysoTracker™ Deep Red (L12492 Termofisher), which can accumulate in acidic compartments such as lysosomes due to proton trapping. For this experiment, LysoTracker media was made with a final concentration of 65 nM in normal cell culture media prior to staining and kept at 37°C in the dark until use. After removal of the original media at the end point of designated treatment, LysoTracker media was added, the cells were incubated for 30 min. Once the Lysotracker media was removed, cells were washed twice with PBS. Add Hoechst (Hoechst media was made with a final concentration of 1 μM in normal cell culture media prior to staining) immediately and incubate for 5 minutes and kept at 37°C in the dark until use. Images were obtained with a Nikon scanning confocal microscope. The number of Lysotracker dots was determined by randomly selecting 3 areas for each sample using Image J software.

3. LysoSensor assay

Measurement of lysosomal pH was performed using LysoSensor™ Yellow/Blue DND-160 (Invitrogen, USA). Cells were seeded onto a 96-well black microplate (1 × 104 per well). LysoSensor staining media was made with Hank's Balanced Salt Solution (HBSS) to a final concentration of 2 μM, which was preheated at 37°C in the dark until staining. After the cell media was removed and cells were washed with PBS, LysoSensor staining media was added (200 μL), and the cells were incubated for 25 min. Following the incubation, the media was removed, and the cells were washed twice with PBS and then left in 100 μL of fresh HBSS and fluorescence was measured quickly. Briefly, after staining with LysoSensor staining media, pH calibration buffer (pH 3.5, 4.5, 5.5, 6.5, 7.5, Bestbio) containing 20 μM Nigericin (MedChemExpress, USA) was added to the cells for 10 min. Then, measurements were acquired according to the method above, and one standard curve was required for each cell type.

4. Cell transfection with adenoviruses

The cells were uniformly seeded in confocal dishes at a density of 3 × 105/ml (2 ml in each dish). Subsequent to the cells from different groups cultured for 5 days, they were transiently transfected with fluorescent mRFP–GFP–LC3 adenovirus (1.58 × 1010 pfu/ml) according to the manufacturer's instructions (Hanbio Technology, Shanghai, China). Images were obtained using a confocal microscope (Nikon AIP, Nikon Corporation, Minato-ku, Tokyo, Japan).

5. Alizarin Red S staining

Cells from different groups were added to the type I collagen (Type I collagen was extracted by acid dissolution [1]) network and incubated at 37°C for the indicated time period. At the designated time period, the cells were stained with Alizarin Red S after fixation with 10% formaldehyde. Excessive dye was removed after 30 min by washing with water. The plates were imaged with a Zeiss microscope (Thorn-wood, NY, USA). Stained areas were measured with the ImageJ software.

6. Extracellular vesicle isolation

For extracellular vesicle collection from medium, the cells were uniformly seeded in petri dishes at a density of 13.7 ×105/ml. After the designated culture period of the tendon fibroblasts in control and the IL-1β and TNF-α medium, the supernatant was

collected and subsequently centrifuged at 10000g for 30 min and 100000g for 70 min. The EVs were isolated from the supernatant by centrifugation at 100000g for 70 min and then washed twice with filtered PBS. The pellets of EVs from medium were resuspended in 200 μl of PBS for NTA, electron microscope assays. The protein content of isolated EVs was measured using the bicinchoninic acid (BCA) Kit (Beyotime BioTech).

7. Western Blot

Cell lysate was prepared using RIPA lysis buffer (Beyotime), and subsequently sonicated to extract tissue proteins. The samples were divided by 10% sodium dodecyl sulfate-polyacrylamide gelelectrophoresis (SDS-PAGE) after which they were transferred to polyvinylidene difluoride membranes (IPVH00010, Millipore). Primary antibodies were used for immunoblotting against the following proteins: anti-CTSD (HUABIO, catalogue number: ET1608-49,), LC3B (Cell Signaling, catalogue number: E5Q2K), LAMP1 (Novus Biologicals, catalogue number: NBP1-77461), CD9 (Abcam, catalogue number: ab125011). After electrophoresis, the polyvinylidene difluoride membranes were incubated with secondary goat anti-mouse IgG horseradish peroxidase-conjugated antibody (Abcam, catalogue number: ab150113). Glyceraldehyde 3-phosphate dehydrogenase anti-GAPDH (HUABIO, catalogue number: ET1702-66) was used as internal control. The stained bands were quantified using image J software.

[1] L. Rittié, *Methods Mol Biol*. **2017**, *1627*, 287-308.
